# Microfluidic and computational study of structural properties and resistance to flow of blood clots under arterial shear

**DOI:** 10.1007/s10237-019-01154-0

**Published:** 2019-05-04

**Authors:** Alexander Y. Mitrophanov, Vijay Govindarajan, Shu Zhu, Ruizhi Li, Yichen Lu, Scott L. Diamond, Jaques Reifman

**Affiliations:** 1 The Henry M. Jackson Foundation for the Advancement of Military Medicine, Inc., Bethesda, MD 20817 USA; 2 Department of Defense Biotechnology High Performance Computing Software Applications Institute, Telemedicine and Advanced Technology Research Center, U.S. Army Medical Research and Materiel Command, Fort Detrick, MD 21702-5012 USA; 3 Institute for Medicine and Engineering, University of Pennsylvania, Philadelphia, PA 19104 USA; 4 Department of Chemical and Biomolecular Engineering, University of Pennsylvania, Philadelphia, PA 19104 USA

**Keywords:** Platelets, Thrombin, Fibrin, Blood flow, Viscous resistance, Axial flow velocity

## Abstract

The ability of a blood clot to modulate blood flow is determined by the clot’s resistance, which depends on its structural features. For a flow with arterial shear, we investigated the characteristic patterns relating to clot shape, size, and composition on the one hand, and its viscous resistance, intraclot axial flow velocity, and shear distributions on the other. We used microfluidic technology to measure the kinetics of platelet, thrombin, and fibrin accumulation at a thrombogenic surface coated with collagen and tissue factor (TF), the key clot-formation trigger. We subsequently utilized the obtained data to perform additional calibration and validation of a detailed computational fluid dynamics model of spatial clot growth under flow. We then ran model simulations to gain insights into the resistance of clots formed under our experimental conditions. We found that increased thrombogenic surface length and TF surface density enhanced the bulk thrombin and fibrin generation in a nonadditive, synergistic way. The height of the platelet deposition domain—and, therefore, clot occlusivity—was rather robust to thrombogenic surface length and TF density variations, but consistently increased with time. Clot viscous resistance was non-uniform and tended to be higher in the fibrin-rich, inner “core” region of the clot. Interestingly, despite intraclot structure and viscous resistance variations, intraclot flow velocity variations were minor compared to the abrupt decrease in flow velocity around the platelet deposition region. Our results shed new light on the connection between the structure of clots under arterial shear and spatiotemporal variations in their resistance to flow.

**Electronic supplementary material:**

The online version of this article (10.1007/s10237-019-01154-0) contains supplementary material, which is available to authorized users.

## Introduction

The formation of blood clots in vivo occurs under flow, which makes blood flow an essential regulator of the clotting process (Casa and Ku 2017; Fogelson and Neeves 2015; Hathcock 2006; Rana and Neeves 2016). Blood clots are formed as a result of complex interactions between the blood vessel wall (which is disrupted or modified at the site of clot formation), blood cells (primarily platelets), and a large biochemical network that predominantly involves the proteins participating, directly or indirectly, in the formation of fibrin—a polymeric protein that provides increased mechanical robustness to the clot (Rana and Neeves 2016). Platelets form the body of the clot and provide catalytic surfaces for the blood coagulation biochemistry (Fogelson and Neeves 2015). Blood flow simultaneously transports these molecular and cellular components to and from the clot-formation region. Understanding and predicting the net outcome of these concurrent processes are challenging due to the spatial and temporal variations in the flow, as well as to the presence of interlinked functional feedback loops and of biochemical reactions that can accelerate or inhibit clot growth. Certain pathologies, such as venous and arterial thrombosis, can lead to persistent changes in local blood flow (Hathcock 2006; Jackson 2011). The need to develop advanced approaches to treat cardiovascular disease necessitates detailed investigations of blood coagulation under flow.

The recent introduction of microfluidic devices has provided enhanced means for in vitro investigations of clot formation in flowing blood (Hosokawa et al. 2011; Muthard et al. 2015; Welsh et al. 2012; Zhu et al. 2015a). The microfluidic channel design, combined with fluorescence microscopy, facilitates the analysis of clot microstructure under physiologically or pathologically relevant conditions. Moreover, experimental parameters, such as the blood-flow rate and the surface concentration of the molecular triggers of clot formation, can be carefully and independently controlled. Accumulation kinetics for platelets, fibrin, and thrombin (the enzyme that converts fibrinogen—the monomeric, soluble precursor protein—to fibrin) can be measured simultaneously with sufficient temporal and spatial resolution.

The use of microfluidic technology in blood research has brought about a new level of knowledge regarding clot structure and dynamics (Colace et al. 2012; Hosokawa et al. 2011; Muthard et al. 2015; Welsh et al. 2012; Zhu et al. 2015a, b, 2016). The structural features of a clot include clot shape and size, considered together with the spatial distributions of the clot’s molecular and cellular constituents, such as coagulation proteins and platelets. However, detailed understanding of the characteristic patterns relating blood clot structure to the clot’s ability to affect the flow—such as the spatial distribution of flow velocity magnitude in the vicinity of the clot as well as within it—remains a challenge. Yet, it is precisely these properties that directly shape the ability of a clot to divert or stop blood flow in bleeding or thrombosis. Given the bidirectional influence between clot structure and resistance, the two should be investigated simultaneously to elucidate their interrelations. The purpose of this study is to gain insights into such interrelations for clots growing under arterial shear. Our interest in arterial-shear flows was motivated by the high prevalence and risk of pathological blood clotting in arteries, which is a major causative factor of cardiovascular disease (Casa and Ku 2017; Jackson 2011; Previtali et al. 2011). Because the magnitude of shear strongly impacts blood coagulation (Ruggeri et al. 1999; Savage et al. 1998; Schneider et al. 2007; Shen et al. 2008), it is conceivable that the characteristic patterns governing the structural features and viscous resistance of blood clots may be shear-dependent and, therefore, different between venous- and arterial-shear flows. We hypothesize that structural properties and resistance of clots are correlated, but can be differentially affected by factors controlling clot-formation initiation.

To investigate this hypothesis, we used a research strategy integrating microfluidic experiments with computational modeling. We utilized a microfluidic device developed by one of us (Colace et al. 2012; Maloney et al. 2010), in which clot formation is initiated by exposure of flowing whole blood to a thrombogenic surface coated with the proteins collagen and tissue factor (TF), which are the same triggers as those initiating clot formation in vivo. Specifically, collagen contributes to platelet adhesion and activation, while TF triggers the biochemical reactions that ultimately lead to the generation of thrombin and fibrin. We used fluorescent antibodies in combination with microscopy to visualize the temporal kinetics of platelet deposition, thrombin formation, and fibrin accumulation. These data were used to calibrate and validate our computational fluid dynamics (CFD) model, which was originally developed to model clot formation under venous-shear flow (Govindarajan et al. 2016). The model reflects the coupling between the local flow properties, blood coagulation biochemistry, and platelet dynamics. This model allowed us to investigate the viscous resistance of the clot, which was not measured in our experiments. Related CFD modeling strategies have been used in blood coagulation research by other authors (Babushkina et al. 2015; Dydek and Chaikof 2016; Jordan and Chaikof 2011; Leiderman and Fogelson 2011, 2013; Ngoepe and Ventikos 2016; Polanczyk et al. 2015; Rugonyi et al. 2010). An advantage of our modeling approach is that it allows one to simulate detailed biochemistry and the platelet- and fibrin-dependent resistance of the growing blood clot, as well as the resultant changes in the surrounding blood flow. While our own work recently addressed both microfluidic (Govindarajan et al. 2018; Zhu et al. 2015b) and mathematical modeling (Govindarajan et al. 2016, 2018) aspects of clot growth under flow, those studies were not focused on the characteristic structure-resistance patterns of clots under arterial shear, which is the main contribution of the present work.

We found that increased thrombogenic surface length and TF surface density synergistically enhance thrombin and fibrin accumulation. As anticipated, clot length was strongly correlated with thrombogenic surface length; in contrast, clot height was more robust to experimental-condition variations. Moreover, our simulations showed that variations in clot composition translate into pronounced viscous-resistance gradients within the clot. However, the resultant variations in axial flow velocity within the clot were minor compared to the abrupt decrease in velocity that defined a rather sharp clot boundary. Our findings shed new light on the factors that control the size, shape, molecular/cellular composition, and resistance of blood clots under arterial shear, which may lead to improved understanding of the molecular mechanisms underlying thrombosis and pathological bleeding.

## Materials and methods

### Subject group

Human sample collection was approved by the University of Pennsylvania Institutional Review Board (Philadelphia, PA) and by the Human Research Protection Office, Office of Research Protections, U.S. Army Medical Research and Materiel Command (Fort Detrick, MD). All blood donors (healthy volunteers, three men and two women) provided informed consent to participate in the study and reported themselves to be free of alcohol use and medication for at least 72 h before blood collection. Donor recruitment, sample collection, and the microfluidic experiments were carried out at the University of Pennsylvania, whereas the computational modeling was performed at the Department of Defense Biotechnology High Performance Computing Software Applications Institute.

### Sample collection and preparation

Samples were collected via venipuncture into corn trypsin inhibitor (CTI, FXIIa inhibitor, 40 μg/mL), which ensured that coagulation initiation by the contact pathway was inhibited during the entire course of the experiments. All microfluidic experiments were initiated within 5 min after phlebotomy. Platelets were labeled with anti-human CD41a antibody (BD Biosciences, CA). To enable simultaneous detection of platelet-associated thrombin during experiments, a thrombin-sensitive platelet-binding sensor (ThS-Ab), synthesized as previously described, was added to blood (1:10 v/v %) before perfusion (Welsh et al. 2012). Fluorescent fibrinogen was added [1 mg/mL stock solution, 1/80 (volume/volume) in whole blood] to measure fibrin accumulation (Life Technologies, Grand Island, NY).

### Microfluidic experiments

**Fig. 1:**
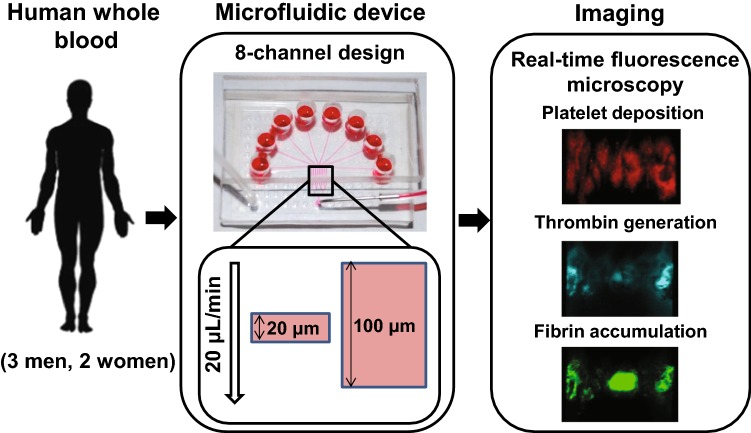
Microfluidic experimental design. Whole blood from five donors (one blood draw per donor) was collected and perfused through an 8-channel microfluidic device. All channels were connected to an outlet with controllable flow rate, which was set to 20 µL/min. Each channel had a rectangular cross section that was 60 µm high and 250 µm wide. The 8 channels were grouped into 4 independent pairs, with one channel in each pair harboring a thrombogenic surface and the other serving to compensate flow. A clot could form only in the channel with a thrombogenic surface; thus, one experiment could yield 4 clots from the same subject growing under the same experimental conditions (i.e., 4 repetitions of the experiment). We used four variants of the microfluidic device that differed in the length of the thrombogenic surface (20 µm or 100 µm in the direction along the length of the channel) and the surface density of TF (~ 0.1 or ~ 2 molecules/µm^2^). During clot growth in the microfluidic device, we used fluorescence microscopy to capture the accumulation of platelets, thrombin, and fibrin at the thrombogenic surface. The resulting images were digitally processed to quantify the fluorescence data

We used an 8-channel microfluidic device, developed by one of us and fabricated as previously described (Govindarajan et al. 2018; Zhu et al. 2015b). The device operated in the pressure relief mode, which allows for physiologically relevant clot growth regimes by preventing possible premature clot washout (Colace et al. 2012). The inlets of the individual channels were held at a constant pressure, and a constant outflow was maintained at the outlet by withdrawing blood through a syringe pump (Harvard Apparatus PHD 2000, Holliston, MA). A particular feature of this study was the choice of a 20 µL/min flow rate, which yielded a target wall shear rate of 1000 s^−1^, a typical value for arterial flows investigated in a previous study by one of us (Colace et al. 2012) (Fig. 1).

Whole blood from volunteers was perfused through the microfluidic device, and clot formation was initiated on the thrombogenic surfaces in the individual microfluidic channels (Figs. 1 and S1). The number of repetitions per experiment was four, because we simultaneously grew and analyzed four clots per blood sample (Fig. 1). We used rectangular thrombogenic surfaces of the same width (250 µm) and two lengths (here referred to as “short” and “long,” respectively): Thus, changes in the surface length directly translated to changes in the surface area. For each length, we used two levels of TF surface density (~ 0.1 and ~ 2 molecules/µm^2^, referred to as “low” and “high,” respectively). The thrombogenic surface length values were 20 and 100 µm for the short and long surfaces, respectively. The surfaces were patterned with collagen and TF (Govindarajan et al. 2018; Zhu et al. 2015b). To that end, glass slides were coated with Sigmacote^®^ (Sigma, St. Louis, MO) and then dried with filtered air. A volume of 5 µL collagen (1 mg/mL, Chrono-log, Havertown, PA) was perfused through two different patterned channels (20 and 100 µm in length) of a microfluidic device to create fibrillar collagen strips, as previously described (Neeves et al. 2008). Lipidated TF was then sorbed to the collagen surface by introducing 5 µL of Dade^®^ Innovin^®^ PT reagent (20 nM stock concentration) to obtain surfaces with the desired TF densities (Zhu et al. 2015b).

Platelet, thrombin, and fibrin accumulation in real time during the experiment was continually monitored and visualized using fluorescence microscopy (IX81, Olympus America Inc., Center Valley, PA, USA). Real-time images were captured with a charge-coupled device camera (Hamamatsu, Bridgewater, NJ, USA) and subsequently analyzed using the ImageJ software from the National Institutes of Health. Figure S2 shows the examples of raw fluorescence data. The spatial distribution of platelet deposition at different time points was measured using confocal microscopy with a disk spinning unit (IX2, Olympus America). Measurements were taken in 50- or 60-s intervals for 400 or 450 s after clot growth initiation. The fluorescence intensity measurements for platelets, thrombin, and fibrin, as well as the platelet profiles, were averaged across donors and repetitions to calculate the overall clotting kinetics and platelet deposition profiles. We used custom-written scripts implemented in MATLAB R2015b (MathWorks, Inc., Natick, MA) to process the fluorescence data (Colace et al. 2012; Govindarajan et al. 2018). We averaged the fluorescence data over the experimental subjects and repetitions. We generated each individual two-dimensional (2-D) platelet deposition profile by averaging the raw fluorescence output across the 250-µm width of the thrombogenic surface. Because our fluorescence reader did not record the absolute position of the thrombogenic surface, the instrument’s frame of reference was different for different clotting events. Therefore, for comparisons and averaging, distinct fluorescence profiles needed to be aligned using a custom-designed algorithm. Individual platelet profiles with spurious platelet accumulation were filtered out. For the retained profiles, we determined the upstream and downstream edges of the region with substantial platelet accumulation and then shifted the profiles to make their centers coincide. For data presentation purposes, the coordinate of the centers for the aligned profiles was arbitrarily set to 300 µm.

### Fully coupled CFD model

As the foundation for our modeling effort, we used the recently developed, detailed 2-D mathematical model of clot growth under venous shear (Govindarajan et al. 2016). The model is a system of partial differential equations that represent the Navier–Stokes equations for the blood flow coupled with the reaction–convection–diffusion equations for four functional platelet phenotypes and nine key proteins in the blood coagulation network (namely, TF, factors II, V, VII, VIII, IX, X, fibrinogen, and tissue factor pathway inhibitor, as well as their numerous complexes and active/inactive forms). An essential element in the model is the additional source term (termed the “Brinkman term”) in the Navier–Stokes equations, which allowed us to reflect the ability of the growing clot to influence the flow via platelet- and fibrin-dependent local viscous resistance (Govindarajan et al. 2016, 2018; Leiderman and Fogelson 2011, 2013). The model implicitly accounted for the presence of red blood cells via enhanced diffusivities for the unbound platelets and biochemical species (Zydney and Colton 1988). Furthermore, we used the Bark–Ku model to represent platelet margination (Bark and Ku 2013). The model equations were solved in a 2-D geometry (Fig. S1) that had been specifically designed to give a consistent 2-D representation of the 3-D microfluidic device (Colace et al. 2012; Govindarajan et al. 2016, 2018).

The continuum-based approach used in our model development represents platelets and biomolecules as time-dependent concentration fields, neglecting the formation of fibrin oligomers and polymerized strands, platelet–fibrin aggregates, and other higher-order microstructure elements. This representation did not allow us to reflect some mechanical properties of clots, such as their viscoelastic characteristics. Nevertheless, this approach was suitable for our purposes because the main focus of our study was to investigate the blood flow and the ability of a clot to modulate it, rather than the mechanics of the clot itself. The ability of the clot to influence the flow was embodied by its resistance to flow (i.e., by its viscous resistance).

**Fig. 2:**
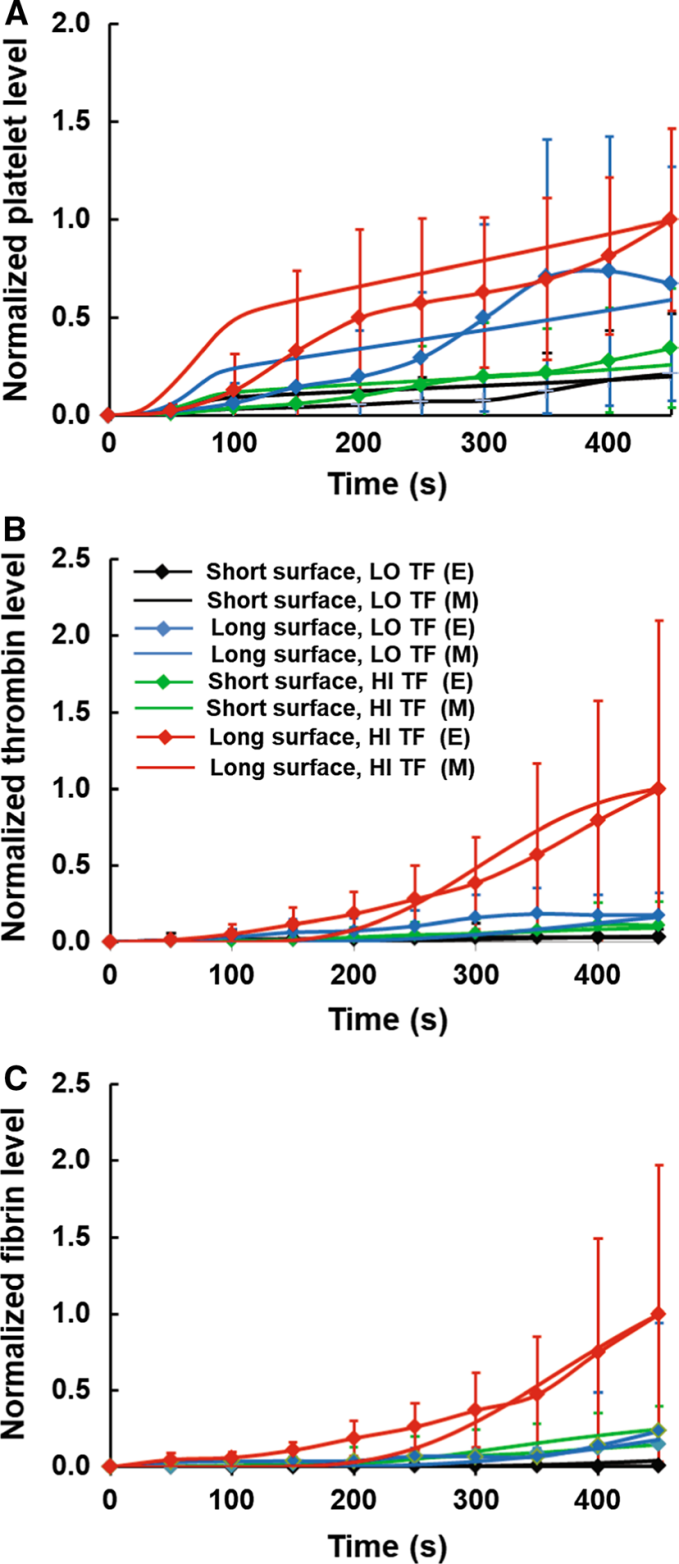
Model-simulated and experimentally measured kinetics of blood clot formation at the thrombogenic surface in the microfluidic device. **a** Platelet deposition, **b** thrombin generation, and **c** fibrin accumulation. Solid lines without markers and with markers designate the fully coupled CFD model simulation results (averages over the flow domain, “M” in the legend) and bulk fluorescence microscopy measurements (“E” in the legend), respectively. For each line, the color reflects the corresponding experimental condition, as explained in the legend [long or short thrombogenic surface, and high (“HI TF”) or low (“LO TF”) TF surface density]. For the plotted experimental data, the markers show data sample means, and the error bars correspond to one standard deviation; the line segments connecting the markers are shown to enhance the visual perception of the data trends. For each of the four experimental conditions, the data (i.e., the measurements for individual clotting events) from different donors (5 donors; one blood draw per donor) and repetitions (4 repetitions) were pooled, resulting in *N* ~ 20. (For some conditions, *N* < 20 because the data for certain repetitions were filtered out so as to retain only the most reliable data points. The excluded data points were compromised due to experimental complications, such as having a chunky fluorescent label during measurement.) The fluorescence levels were measured in arbitrary units, but the CFD model outputs were in absolute concentration units. Therefore, for proper comparisons, the model outputs and the experimental data needed to be normalized. For platelet deposition (subplot **a**), the experimental data were normalized by dividing the data by their maximum average value. The corresponding model-generated time courses were normalized by dividing the model outputs by their maximum value. The thrombin (subplot **b**) and fibrin (subplot **c**) data were normalized in a similar way. In each subplot, the differences between the experimentally measured output level for the long thrombogenic surface with high TF surface density and the corresponding levels for the remaining three conditions were statistically significant at 450 s

The boundary and initial conditions for the equations reflected the experimental conditions chosen for our microfluidic measurements. The main model inputs were the thrombogenic surface length and the surface density of TF. The main outputs were the time- and location-dependent concentrations of platelets, thrombin, and fibrin accumulated at the thrombogenic surface. All input and output concentrations in the model were specified in absolute units. However, for the purposes of comparisons with the experimental data (reported in arbitrary units), the model outputs (and also the experimental data) needed to be normalized to the appropriate maximum values (see, e.g., Fig. 2). While this approach has limitations, it is common in biomedical research and has been previously used in a variety of contexts [e.g., (Chen et al. 2009; Klipp et al. 2005)].

The coagulation protein concentrations and other parameters not explicitly modified to reflect our experimental setup had their default values, which reflected the typical, average values representing an “average” human subject with no blood coagulation abnormalities. This is consistent with the aim of our modeling, which was to analyze the typical trends in the structural and resistance changes of blood clots. The model was implemented and solved in the CFD package FLUENT v. 17.1 (ANSYS, Inc., Canonsburg, PA) (Govindarajan et al. 2018). One run of the model for a given set of conditions and parameter values took ~ 1.5 days, using 64 cores in a high-performance computing platform. We refer the reader to Supplementary Material for the full list of equations and boundary conditions, as well as the default model parameter values.

### Clot “core” and “shell” modeling

**Fig. 3:**
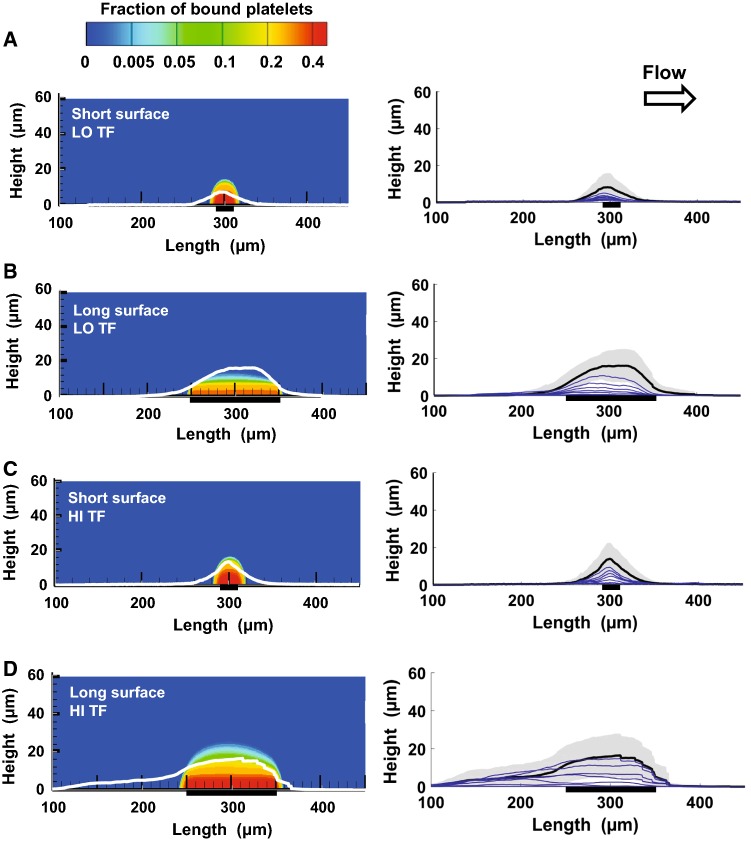
Computational model predictions and confocal microscopy data on the spatial distribution of platelet deposition domains. Flow direction is from left to right. The subplots **a**–**d** correspond to the four analyzed combinations of conditions, as indicated in the figure [long or short thrombogenic surface, and high (“HI TF”) or low (“LO TF”) TF surface density]. In each subplot, the left panel shows the model-simulated platelet deposition at 400 s as a density plot, with different colors representing different values of the fraction of bound platelets. The superimposed white lines show the mean platelet deposition domain boundary at 400 s based on the experimental data. For each of the four experimental conditions, the data (i.e., the measurements for individual clotting events) from different donors (5 donors; one blood draw per donor) and repetitions (4 repetitions) were pooled, resulting in *N* ~ 20. (For some conditions, *N* < 20 because the data for some repetitions were filtered out so as to retain only the most reliable data points). At the middle of the thrombogenic surface (at 400 s), the difference in the experimental data between the long and short thrombogenic surface conditions was statistically significant for both TF surface density values. At the same time and location, the difference in the experimental data between the high and low TF surface density was significant for the long thrombogenic surface but not for the short one. In each subplot, the right panel shows experimental data on platelet deposition kinetics. The concave black line is the same mean platelet domain boundary line as that shown (in white) in the left panel. The gray-shaded area corresponds to ± one standard deviation from the mean platelet profile shown by the black line. The thin blue lines (bottom to top) show mean platelet deposition profiles corresponding to times from 50 to 350 s in 50-s increments. In each subplot, the thick horizontal black lines indicate the location of the thrombogenic surface

The core and shell boundaries were obtained from the simulations performed using the fully coupled CFD model described in the previous subsection. First, we visualized the model-simulated spatial distributions of the deposited platelets (see the third subsection of Results section). The boundaries of the platelet deposition domains were determined by visual inspection, and the resulting regions were designated as the clot shell regions. Then, the edges of the fibrin accumulation domains were determined by applying a 1-nM fibrin concentration threshold, which had yielded an accurate approximation of the fibrin deposition domain boundary in comparison with experimental data [Fig. 3c in (Govindarajan et al. 2016)]. The resulting regions were designated as the clot core regions. Subsequently, the core and shell regions were superimposed.

### Flow simulations with experimentally measured, averaged clot shapes

The platelet deposition profiles obtained using confocal microscopy were averaged across experimental measurement repetitions and across donors for each of the four experimental conditions (namely, 2 thrombogenic length values × 2 TF surface density values = 4 distinct conditions). The boundaries of the platelet profiles thus averaged (one profile per each of the four considered experimental conditions) were regarded as the *shell* region boundaries of porous clots. Before the averaging, all individual profiles corresponding to a given experimental condition were aligned using custom-written MATLAB scripts. This alignment involved shifting the profiles along the length coordinate so as to position the profile centers at the center of the thrombogenic surface.

As a first step to define the corresponding *core* region boundaries, we took the model-simulated core regions (described in the previous subsection) and rescaled their height and width to embed them within the clot shell regions derived from our confocal data on platelet deposition. We performed this rescaling so that the core-to-shell ratios for the height and the length were the same as those for the cores and shells simulated using the fully coupled CFD model under the same conditions. We chose to perform the rescaling in this way to ascertain the consistency of our core–shell definitions with our analogous simulations for a venous-shear flow (Govindarajan et al. 2018), as well as with our simulations using the fully coupled CFD model. Then, we assigned to the resultant cores and shells distinct viscous resistance values, which were the spatially averaged core and shell viscous resistance values from the simulations with the fully coupled CFD model (under the same conditions). Finally, we simulated blood flow through these experimentally derived porous core–shell structures by solving the Navier–Stokes equations (in FLUENT v. 17.1). One simulation took ~ 1 h, using 64 cores in a high-performance computing platform. All initial and boundary conditions, as well as simulation convergence criteria for these simulations, were the same as those for our fully coupled CFD model.

### Statistical analysis of experimental data

Fluorescence measurements from different donors and experimental repetitions formed the data samples that we analyzed using statistical methods. The samples were tested for normality using the Jarque–Bera test (Jarque and Bera 1987). Normal samples were compared using the unpaired *t* test, and non-normal samples were compared using the rank-sum test (Glantz 2005). In cases of multiple comparisons, we used the Bonferroni correction for *P* value adjustment (Glantz 2005). *P* ≤ 0.05 was considered statistically significant. To fully utilize the available data in our sample comparisons between experimental conditions, we pooled data points from different donors and different repetitions—corresponding to a single experimental condition—into a single data sample. The main general issue with data pooling is the possibility of non-independence for distinct data points (Jenkins 2002). However, in our case, distinct clotting events could be regarded as statistically independent, because, due to our experimental design, the occurrence of a clotting event did not affect the probabilities of other such events.

## Results

### Model calibration and validation using kinetic fluorescence data

To assess the prediction accuracy of our fully coupled CFD model, we compared the model outputs with our fluorescence data on the kinetics of platelet deposition, thrombin generation, and fibrin accumulation. This initial assessment showed that the CFD model systematically underpredicted the experimentally measured kinetics, suggesting that it needed additional calibration. Existing data suggest increased platelet adhesion and aggregation under arterial shear due to the involvement of von Willebrand factor (Ruggeri et al. 1999; Savage et al. 1998; Schneider et al. 2007). In accord with this evidence, we expected that increasing the platelet adhesion rate in the model would enhance model accuracy for platelet deposition and, consequently, for thrombin generation and fibrin accumulation. We performed heuristic tuning of this rate to improve the correspondence between the model predictions and the fluorescence data for platelets, and used the thrombin and fibrin fluorescence data as the validation data set. This was the only parameter we adjusted, out of the 100 parameters in the model (see the tables in Supplementary Material for the complete parameter information). The final, post-calibration value of the platelet adhesion rate was fourfold higher than its pre-calibration value. The outputs of the calibrated model are plotted in Fig. 2 against the fluorescence data. The calibrated model captured the average trends in the data with sufficient fidelity.

### Increased thrombogenic surface length and TF surface density synergistically enhance thrombin and fibrin accumulation

Our CFD model captured, and our experimental results confirmed, the following characteristic patterns. As expected, the levels of platelets, thrombin, and fibrin were the highest for the long thrombogenic surface with high surface density of TF, and lowest for the short surface with low TF density (Fig. 2). Increasing the thrombogenic surface length or TF surface density *separately* had a moderate effect of comparable magnitude on the thrombin and fibrin kinetics (Fig. 2b, c). In contrast, *simultaneously* increasing these experimental parameters dramatically (> 20-fold at 450 s) increased these two outputs, which implies nonadditive, synergistic acceleration (Fig. 2b, c, red lines). For platelets, increasing the thrombogenic surface length and TF surface density also led to heightened output levels, but the synergism was, overall, less pronounced (Fig. 2a). This is consistent with the notion that the influence of TF on thrombin generation is more direct than on platelet accumulation.

The congruency of the synergistic effects for thrombin and fibrin is consistent with the role of thrombin as the primary factor (specifically, the producing enzyme) behind fibrin accumulation. The synergistic effect for thrombin can be understood by noticing that the total abundance of TF is a product of TF surface density and the area of the thrombogenic surface, which implies a multiplicative—rather than additive—dependence of the thrombin output on these two factors. Yet, due to the complexity of the blood coagulation system, intuition alone would not be sufficient to make a reliable prediction regarding the magnitude of this effect. Taken together, our results demonstrate that the fully coupled CFD model is capable of predicting the synergistic interaction between the distinct aspects of TF localization.

### Clot height monotonically increases with time, but is robust to variations in TF localization

CFD simulations using our calibrated model predicted that the peak height of the platelet deposition domain at 400 s should only weakly depend on the experimental conditions (Fig. 3, left panel). The experimental data on platelet deposition at 400 s were generally consistent with this prediction (Fig. 3, left panel). Although the quantitative accuracy of the platelet deposition domain predictions varied between experimental conditions, the model predictions for clot height were within one standard deviation of the corresponding experimental mean values. The model-predicted peak heights varied between 16 and 19 µm for the majority of conditions, except the long thrombogenic surface with high TF density, for which the peak was ~ 24 µm (Fig. 3, left panel). The experimentally determined mean peak height was smallest (~ 7.5 µm) for the short thrombogenic surface with low TF surface density (Fig. 3a). For the other three conditions, it varied in the ~ 13- to 16-µm interval (Fig. 3b–d). As expected, the length of the platelet deposition area at 400 s positively correlated with the length of the thrombogenic surface, both in the model predictions and in the experimental results (Fig. 3, left panel). As a consequence, the same pattern also held for the area under the mean platelet deposition profile, reflecting the total size of the platelet deposition domain.

The general patterns described above for 400 s were in accord with the kinetics of clot growth reflected by the mean platelet profiles experimentally measured at earlier time points (Fig. 3, right panel). These profiles show smooth, monotonic growth of the platelet deposition domain over time, with wider platelet profiles corresponding to longer thrombogenic surfaces. Interestingly, for most experimental conditions, the vertical spaces between the profiles at later time points increase, suggesting the possibility of platelet deposition acceleration with time (Fig. 3a–c, right panel).

Taken together, our data indicate that the overall clot size at a wall shear rate of 1000 s^−1^ is determined predominantly by the length (and, therefore, area) of the thrombogenic surface. In contrast, the platelet deposition domain height—and, therefore, clot occlusivity (i.e., the ratio of the clot height to the microfluidic channel height)—appears to be more robust to thrombogenic surface area and TF surface density variations. Yet, there is a trend toward larger and taller clots for the large thrombogenic surface with high surface density of TF (Fig. 3d), which is consistent with the bulk kinetics of platelet accumulation (Fig. 2a).

### Model-simulated, condition-dependent clot structure produces uniform regions of reduced flow velocity

**Fig. 4:**
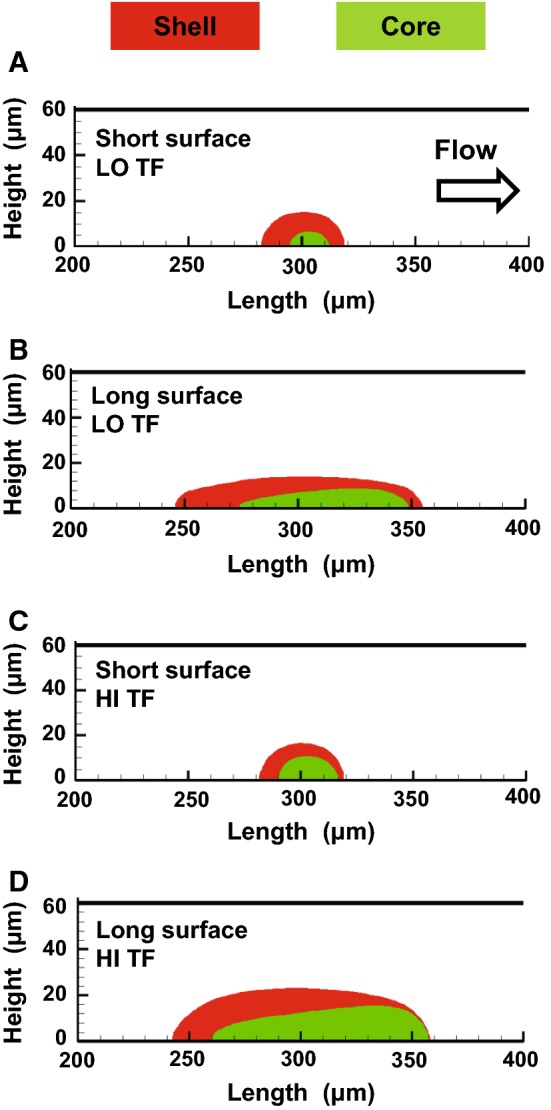
Core and shell regions at 400 s simulated using our fully coupled CFD model. Flow direction is from left to right. The subplots **a***–***d** correspond to the four analyzed combinations of conditions, as indicated in the figure [long or short thrombogenic surface, and high (“HI TF”) or low (“LO TF”) TF surface density]. The boundaries of the core and shell regions were computed as described in Sect. “2”. For each subplot, the center of the thrombogenic surface is at 300 µm

To investigate the inner structure of blood clots, we used our fully coupled CFD model to simulate the formation of the core and shell regions, as described in Sect. “2”. The core–shell architecture reflects the dominant spatial features of the molecular and cellular structure of the clot. We wished to investigate this architecture because the fibrin-rich core and platelet shell have been recently recognized as fundamental structural and functional elements of blood clots both in vitro (in microfluidic experiments) (Muthard et al. 2015) and in vivo (Stalker et al. 2013). At 400 s, as expected, the shell region of the clot fully surrounded the core region (Fig. 4). Because, by construction, the shell regions approximated the model-simulated outer contours of the platelet deposition domains (shown in Fig. 3, left panel), the size of these regions was defined predominantly by the thrombogenic surface length (Fig. 4). In contrast, the size of the fibrin-enriched core regions was noticeably influenced not only by the thrombogenic surface length, but also by TF surface density (Fig. 4). This is consistent with the key role TF plays in the activation of thrombin generation and, therefore, fibrin accumulation. Interestingly, although the shell region was symmetric with respect to the middle of the thrombogenic surface, the model-simulated fibrin-rich core region was visibly shifted downstream, which is in accord with previous experimental work (Muthard et al. 2015).

**Fig. 5:**
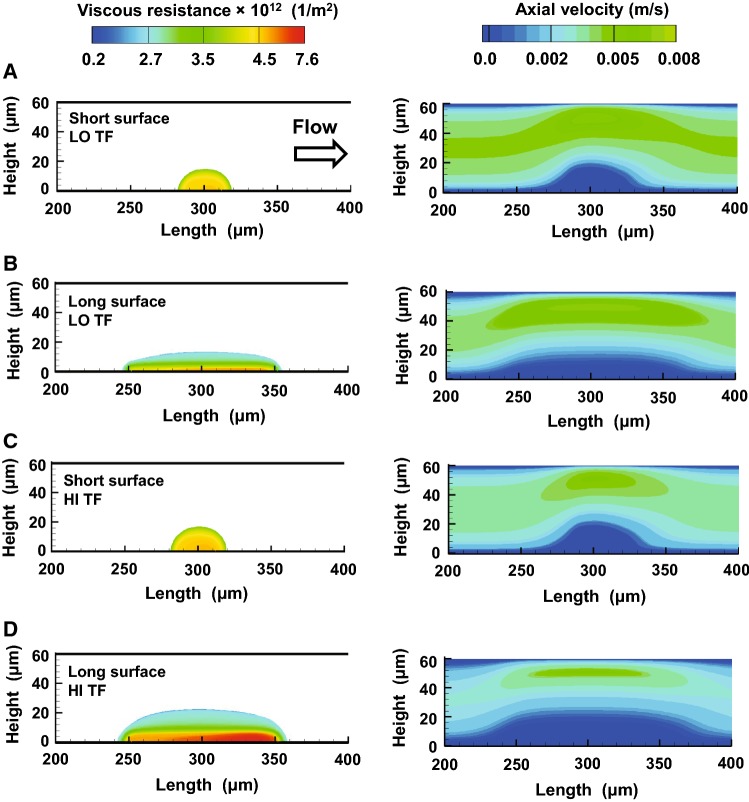
The ability of the clots to affect the flow at 400 s simulated using our fully coupled CFD model. Flow direction is from left to right. The subplots **a**–**d** correspond to the four analyzed combinations of conditions, as indicated in the figure [long or short thrombogenic surface, and high (“HI TF”) or low (“LO TF”) TF surface density]. In each subplot, the left and the right panels show the viscous resistance and blood axial flow velocity, respectively. In the left panel, the shapes of the clots correspond to the model-simulated clot shell regions (i.e., platelet deposition domains) shown in Fig. 4. For each panel in the figure, the center of the thrombogenic surface is at 300 µm

The model-simulated spatial distribution of viscous resistance within the clot core–shell regions was generally consistent with the core–shell architecture and was characterized by a transition between the lower-resistance outer regions and the higher-resistance inner regions (Fig. 5, left panel). Yet, the shape and thickness of these regions (Fig. 5, left panel) were somewhat different from those predicted by the core–shell analysis (Fig. 4). In most cases, the overall shape and location of the high-resistance region appeared more symmetrical around the thrombogenic surface center (Fig. 5a–c, left panel) than those for the corresponding fibrin-enriched core region (Fig. 4a–c). Perhaps surprisingly, the low-resistance outer layer was consistently and considerably thinner for the short thrombogenic surface (Fig. 5a, c, left panel) than for the long one (Fig. 5b, d, left panel).

The clot viscous resistance values for the long thrombogenic surface demonstrated significant spatial variation across the entire clot domain (Fig. 5b, d, left panel), suggesting that the resultant axial flow velocity would follow the same variation pattern. Yet, compared to the velocity outside of the clot domain, the flow velocity within the clot (i.e., intraclot flow velocity) under all considered conditions was uniformly low and was characterized by a somewhat sharp transition at the boundary (Fig. 5, right panel, blue regions). This result suggests that the extent of velocity variation within the clot is considerably less than the velocity variations outside of the clot (i.e., in the channel lumen). The lengths of the low-velocity domains exceeded those of the corresponding clot shell areas, extending both upstream and downstream of the thrombogenic surface (Fig. 5, right panel). Taken together, our simulations suggest that the height of the low-velocity regions remains approximately constant under the considered experimental conditions (at 400 s), and the lengths of these regions are larger for the longer thrombogenic surface.

### Axial flow velocity varies predominantly in the vertical direction for the experimentally measured clot shapes

**Fig. 6:**
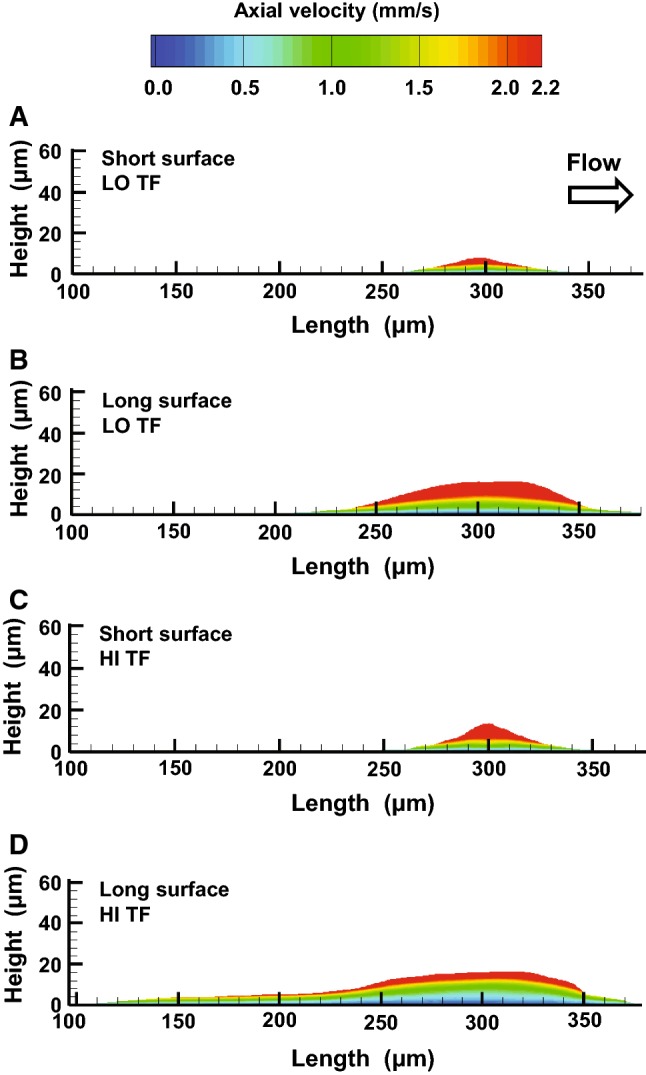
Computationally simulated spatial distribution of axial flow velocity for the experimentally measured, averaged clot shapes at 400 s. Flow direction is from left to right. The subplots **a**–**d** correspond to the four analyzed combinations of conditions, as indicated in the figure [long or short thrombogenic surface, and high (“HI TF”) or low (“LO TF”) TF surface density]. In each subplot, the clot shape is given by the average platelet deposition profile (also shown in Fig. 3). Each clot region was subdivided into the core and shell regions, as described in Sect. “2”. These regions had specific viscous resistance characteristics (based on the simulation results in Fig. 5, left panel), which defined the axial velocity of the blood flow through the clot. To calculate the axial flow velocity, we solved the Navier–Stokes equations as described in Materials and Methods section. For each subplot in the figure, the center of the thrombogenic surface is at 300 µm

Figure 5 (right panel) shows essentially no variation in the intraclot velocity (dark blue areas in the figures). Yet, this lack of variation is relative and exists in the context of velocity-level variations both inside and outside of the growing clot. Consequently, it should be expected that the velocity levels inside the clot actually do vary, and these variations become more visible when *only* the intraclot velocity field is plotted. Specifically, based on the core–shell clot architecture (Fig. 4) and the corresponding viscous resistance distributions from our fully coupled CFD model (Fig. 5, left panel), one can anticipate the existence of considerable vertical intraclot axial velocity gradients. To verify the generality of this notion, we tested it in a somewhat different (yet related) context. Specifically, we confirmed it in simulations of blood flow through porous bodies that corresponded to experimentally derived, averaged platelet deposition domains (see Materials and Methods for the simulation strategy). The advantage of this setup is a realistic representation of the platelet deposition domain surface, where flow velocity is the highest (compared to the inner regions of the clot). In those simulations, the velocity distributions at 400 s were qualitatively and quantitatively similar across experimental conditions; as expected, the highest and the lowest velocities were at the top and at the bottom, respectively, of the platelet deposition profiles (Fig. 6). Interestingly, there was little velocity variation in the horizontal direction inside any of the simulated platelet clots (Fig. 6), despite the presence (by construction) of two distinct zones with different viscous resistance levels (i.e., the core and the shell).

## Discussion

During thrombosis—i.e., when a clot is formed inside of an intact blood vessel—the ability of the clot to resist blood flow is the cause of pathological effects (Casa and Ku 2017; Hathcock 2006; Jackson 2011). The goal of therapeutic interventions is to control this ability, which necessitates in-depth investigations of the factors that shape it. Here, we used microfluidic experiments and computational modeling to investigate the relationships that characterize the structural features of blood clots and their resistance to blood flow at a typical arterial wall shear rate (1000 s^−1^). The particular benefit of computational modeling is that it allowed us to study the resistance of clots, which was not feasible with our experimental approach. First, we found that increased thrombogenic surface length and TF surface density accelerated thrombin and fibrin accumulation in a synergistic fashion (Fig. 2). Second, we established that, under the considered experimental conditions, the platelet accumulation domain size was—expectedly—increased for the longer thrombogenic surface, but was—rather unexpectedly—robust to TF surface density variations (Fig. 3). The dependence of the platelet deposition domain height (at a given time) on the considered experimental conditions was, overall, less pronounced (Fig. 3). Third, our simulations suggested that platelet deposition domains define regions of uniformly low axial flow velocity. These regions have sufficiently sharp boundaries and strongly depend on the thrombogenic surface length (Fig. 5, right panel). Finally, our computational results demonstrated that the intraclot axial velocity varies predominantly in the vertical direction (Fig. 6).

The mathematical modeling framework that we used here had been introduced in our recent work (Govindarajan et al. 2016), which was focused on model development (using published data) and on examining the effects of blood dilution and clotting factor supplementation on blood clotting under venous-shear flow. This modeling framework was further validated with newly generated data on platelet, thrombin, and fibrin accumulation in our subsequent work (Govindarajan et al. 2018), whose focus was on the effects of TF localization under a venous-shear (100 s^−1^) flow, similar to the value (200 s^−1^) considered in (Govindarajan et al. 2016). In the present study, we analyzed a substantially different situation characterized by a shear rate that was fivefold–tenfold higher. It is known that high shear can strongly impact the behavior of biomolecules and cells in whole blood, as exemplified by the shear-dependent behavior of von Willebrand factor and by shear-induced platelet activation (Casa and Ku 2017). Interestingly, validation of our simulation results on newly generated experimental data demonstrated that fine-tuning only one parameter (out of the 100 parameters in the model) was sufficient to considerably improve the accuracy of computational predictions of clot-formation kinetics. We used our computational model, thus adjusted, to obtain insights into the complex relationship between the structure of a clot and its ability to resist—and thereby redirect—blood flow.

The collagen and TF exposed at the injury site or at the site of atherosclerotic plaque rupture directly influence thrombin and fibrin generation and, as a consequence, platelet accumulation, which in turn further modulates the biochemical network of clot formation. Moreover, pathological conditions, such as hyperfibrinogenemia, can directly affect coagulation factor availability and, therefore, clot properties, leading to disease (Machlus et al. 2011). Furthermore, pharmacological interventions aimed at controlling blood coagulation target either the biochemical [e.g., tenecteplase, a widely used fibrinolytic agent (Davydov and Cheng 2001)] or cellular [e.g., aspirin, which is a well-known platelet aggregation inhibitor (Patrono 1994)] components of blood clots. Thus, the primary aspect of the clot that is impacted directly by molecular factors is its structure. Therefore, knowledge of the relationships between clot structure and resistance properties is a prerequisite for understanding the molecular-level control of the clot’s ability to resist blood flow.

The inner composition of blood clots is heterogeneous, as exemplified by our analysis of their core–shell architecture (Fig. 4). Yet, the axial flow velocity distribution inside the clot—compared to that of the clot surroundings—is remarkably uniform (i.e., there is little variation in the intraclot axial velocity; Fig. 5, blue areas in the right panel). Whereas the extent of this uniformity might change at later times, at 400 s it reflects a rather mature stage of clot formation. At earlier times, the uniformly low velocity area is expected to change by progressively increasing in height, similarly to a venous-shear flow (Govindarajan et al. 2016). These results are in accord with simulations using a different approach, which demonstrated that, beyond a certain threshold size, velocity inside the clot does not depend on its size (Tomaiuolo et al. 2014). Functionally, the local reduction in flow velocity may be regarded as the main, integral outcome of clot formation, and a direct reflection of clot-dependent blood-flow modulation. The homogeneity and somewhat sharp boundaries of the reduced-velocity regions, which correlate with the platelet deposition domains, are consistent with the notion that the platelet deposition domain boundary defines the overall shape and size of the clot (Govindarajan et al. 2016).

Our results suggest that the “horizontal” size of the reduced-velocity region (i.e., the length parallel to the blood flow) is determined primarily by the length of the thrombogenic surface, i.e., the size of the pathologically impacted region of the vessel wall. In contrast, the reduced sensitivity of the clot height to experimental condition variations (Figs. 3, 5, right panels) suggests that the “vertical” size (and hence occlusivity) can be similar for both wide and narrow clots. This reduced sensitivity is likely due to platelet shedding from the clot surface, which would increase with the increasing degree of occlusion during clot growth. (Whereas abrupt reduction in clot size due to embolization of larger pieces of clot material could also be possible, our data did not suggest that this was a frequent occurrence.) Because the platelet deposition domains show steady growth in occlusivity with time (Fig. 3, right panel), the time elapsed after clotting initiation—rather than the strength of the signal initiating clot growth—appears to be the main factor that determines clot occlusivity.

The detected strong positive dependence of the clot size on the thrombogenic surface length (and, therefore, on its surface area; Figs. 2a, 3) is intuitively anticipated and consistent with recent results for venous-shear flow (Govindarajan et al. 2018). However, this result could not be directly predicted based on intuition alone, because recent experimental studies for venous-shear flows suggested that the dependence of clot size on thrombogenic surface length may not always be monotonic (Govindarajan et al. 2018; Zhu et al. 2016). This notion of non-monotonicity is in accord with the additional arterial-shear experiments that we performed for a 1000-µm-long thrombogenic surface. In those experiments, clot formation on the 1000-µm surface was considerably less pronounced compared to our 100-µm-long surface—a trend that is puzzling and warrants further investigation. It is also necessary to point out that our study focused only on rather low average degrees of occlusion (experimental averages not exceeding ~ 30%; Fig. 3, left panels). Yet, a major advantage of our microfluidic methodology (specifically, of its pressure relief mode) is its ability to support clot growth to almost occlusion (Colace et al. 2012). It would be insightful, in a future study following our approach, to focus specifically on the characteristics of high-occlusivity clots.

This study has limitations. First, the microfluidic device only provides an approximation to the blood flow and clot-formation conditions that exist in vivo. Indeed, it does not reflect, for example, blood vessel diameter changes and the active involvement of endothelial cells during clot formation. Yet, it allows for focused experiments, in which the relevant parameters are controlled independently and with a reasonable degree of accuracy. This makes it possible to tease out the individual contributions of distinct factors to the overall process of clot formation despite the variability in the microfluidic assay measurements (Neeves et al. 2013). Second, even after additional calibration, our computational model did not capture all the nuances of the experimental data (Figs. 2, 3, left panel). This is understandable because our computational modeling effort, including our model calibration strategy, was focused predominantly on major trends rather than nuances. Third, our 2-D computational model, which represents platelets as continuous spatial distributions of tracer particles, gives a simplified representation of the clot-formation process. Moreover, more detailed computational models of blood coagulation reflecting in vitro systems without flow can contain a much more in-depth representation of the relevant biochemistry than the model we used in this work (Mitrophanov et al. 2014). Yet, recent work by us (Govindarajan et al. 2016, 2018) and others (Babushkina et al. 2015; Dydek and Chaikof 2016; Jordan and Chaikof 2011; Leiderman and Fogelson 2011, 2013) suggests that our 2-D modeling approach achieves a satisfactory balance between accuracy and computational expediency, and is sufficient for many applications. Furthermore, our decision to implement our model in FLUENT provides convenient opportunities for further improvements, such as incorporation of more detailed biochemistry and direct modeling of clot formation in 3-D geometries. We are pursuing such improvements in our ongoing work. Finally, our analysis has been performed for a group of healthy subjects, and the results may have been different for various disease conditions. Yet, both our computational (Govindarajan et al. 2016) and experimental (Li et al. 2016) approaches have been applied to pathological blood coagulation. Therefore, our study design can be directly adapted to settings where blood clotting in healthy volunteers is compared to that in patients with a blood coagulation disorder.

Taken together, our results support our main hypothesis that the structure and resistance of a growing clot are correlated, but can differ in their regulation by the distinct aspects of TF localization. Our work demonstrates how experimental measurements and computational simulations can complement each other to provide new insights into clot properties. If this strategy is applied to experimental studies mimicking arterial disease conditions, then our CFD model or its future improved versions may potentially be used as a “virtual test bed” to perform efficient screening for promising drug candidates and targets to treat blood-clotting disorders.

## Electronic supplementary material

Below is the link to the electronic supplementary material.

Supplementary material 1 (PDF 1234 kb)
