# Supplementary material for: Microfluidic and computational study of structural properties and resistance to flow of blood clots under arterial shear
Source: Biomech Model Mechanobiol. 2019 May 4;18(5):1461–74. doi: 10.1007/s10237-019-01154-0 (PMC6748893; doi:10.1007/s10237-019-01154-0)
Supplement: Supplementary file 1 — Supplementary material 1 (PDF 1234 kb) [file 10237_2019_1154_MOESM1_ESM.pdf]

SUPPLEMENTARY MATERIAL

Microfluidic and computational study of structural properties and resistance to flow of blood clots under arterial shear

Alexander Y. Mitrophanov, Vijay Govindarajan, Shu Zhu, Ruizhi Li, Yichen Lu,
Scott L. Diamond, and Jaques Reifman

MATHEMATICAL MODEL DESCRIPTION

Fluid flow and related equations

In our model, the Navier-Stokes equations for the fluid flow are coupled (and solved together) with the convection-diffusion-reaction equations for the platelets and individual biochemical species. The latter equations have the following general form:

$$\frac{\partial C_i}{\partial t} = -\nabla \cdot (\vec{u} C_i) + \nabla \cdot (D_i \nabla C_i) + R_i \,, \tag{S1}$$

where C_i is the concentration of species i, $\vec{u}$ is the blood flow velocity vector, D_i is the individual species diffusivity, and R_i is the reaction source term. To reflect the effective diffusivity of platelets and plasma proteins due to the presence of red blood cell (RBC) motion, we used the enhanced diffusivity model of Zydney and Colton (1):

$$D_i^e = D_i^b + k d_{rbc}^2 \phi (1-\phi)^\eta \dot{\gamma} \,, \tag{S2}$$

where D_i^e and D_i^b denote the enhanced diffusivity and Brownian diffusivity, respectively, of species i, k and η are empirical constants, d_{rbc} denotes the RBC diameter, ϕ is the local hematocrit, and $\dot{\gamma}$ is the local shear rate. Different enhanced diffusivities are denoted by "D" in Eqs. S8-S62. To obtain their numerical values for different equations, one should take the Brownian diffusivities from Table S1, modify them according to Eq. (S2), and use the resultant values in the Eqs. S8-S62 in accordance with the diffusivities' subscripts (e.g., D_{fibrin}^b from Table S1 should be used in Eq. S54 for fibrin, and $D_{proteins}^b$ should be used for all other proteins).

For the inlet hematocrit profile, we used the "blunt" profile, as previously described (2,3). The effect of clot growth on the blood flow was modeled as an increase in local viscous resistance due to platelet deposition and fibrin accumulation. The growing clot was modeled as a porous medium, whose permeability depends on the fraction of deposited platelets and the concentration of fibrin protomers (2). We modified the standard incompressible Navier-Stokes equations by incorporating additional source term in the momentum equation:

$$\nabla \cdot (\vec{u}) = 0 \,; \tag{S3}$$

$$\rho \left(\frac{\partial \vec{u}}{\partial t} + (\vec{u} \cdot \nabla) \vec{u} \right) = -\nabla p + \mu \Delta \vec{u} - \frac{\mu}{K_t(\vec{x})} \vec{u} \,, \tag{S4}$$

where ρ, $\vec{u}$, p, and μ denote the blood density, fluid velocity vector, fluid pressure, and the dynamic viscosity of blood, respectively. The term $\mu / K_t(\vec{x})$ in Eq. S4 is the Brinkman term, where $K_t(\vec{x})$ denotes the permeability of a porous medium, and $1/K_t(\vec{x})$ represents its viscous resistance. The viscous resistance is imparted by the deposited platelets and fibrin, and their contributions are added:

$$\frac{1}{K_t} = \frac{1}{K_p} + \frac{1}{K_f} \,, \tag{S5}$$

where K_p and K_f denote the permeabilities of the deposited platelet mass and the fibrin mass, respectively. The contribution of the platelets to the total clot viscous resistance is given by the following formula (2,4):

$$\frac{1}{K_p} = \alpha_{\max}\left(\frac{(\phi^b)^2}{(\phi_o^b)^2 + (\phi^b)^2}\right), \qquad \text{(S6)}$$

where ϕ^b denotes the fraction of bound platelets (i.e., the ratio of bound platelets to the maximum possible platelet density), and ϕ_0^b is a constant. The contribution of fibrin to the clot's viscous resistance is computed using the Davies equation (5):

$$\frac{1}{K_f} = \frac{16\phi_f^{1.5}(1+56\phi_f^3)}{a_f^2}, \qquad \text{(S7)}$$

where a_f denotes the fiber radius and ϕ_f is the ratio of the fibrin concentration to that of fibrinogen.

Platelet margination and interactions

Our model has four platelet types: *1*) mobile unactivated (which are unbound and remain in their resting state if there are no external stimuli), *2*) mobile activated (which are unbound and have been activated by the action of thrombin and/or ADP), *3*) platelet-bound activated (mobile activated platelets that adhere to nearby bound platelets), and *4*) subendothelium-bound activated (activated by collagen exposure) (2,4). The densities (i.e., volume concentrations) of these platelet types are denoted by $P^{m,u}$, $P^{m,a}$, $P^{b,a}$, and $P^{se,a}$, respectively. Equations S8-S12 (below in this Supplementary Material) describe the platelet interactions. The bound platelet fraction, equal to $(P^{b,a} + P^{se,a})/P_{\max}$, is the quantity that we plotted in our figures representing platelet deposition (see the main text of the paper). In this expression, $P_{\max}$ is the maximum platelet density equal to 1.0×10^8 platelets/mm^3 (2).

The mobile platelet species marginate due the presence of RBCs, and this leads to their increased concentration at the microfluidic channel walls. To model this phenomenon, we implemented the platelet margination model proposed by Bark and Ku (6). The platelet margination model is embedded in the flux term $\vec{u}C_p - D_p^e \nabla \psi^{m,u(a)}$ in Eqs. S8 and S9. The first term, $\vec{u}C_p$, represents convection of the platelet species due to blood flow, and the field potential in the second term, $\nabla \psi^{m,u(a)} = C_p \nabla(\phi) + \phi \nabla(C_p)$, allows platelets to drift based on the local hematocrit level, ϕ (2,6). C_p is the platelet species concentration, and D_p^e is the enhanced diffusivity. To capture the hindrance caused by the growing thrombus on the mobile platelet species (Eqs. S8 and S9), Leiderman and Fogelson (4) introduced a weight function, $W(\phi^T) = \tanh(\pi(1-\phi^T))$, which was also implemented in our model (2). This function

gradually decreases the ability of the mobile platelets to move, until the total platelet fraction, ϕ^T (ratio of the sum of all the platelet species to the maximum platelet density), reaches a threshold value of 0.5, beyond which the platelet mobility reduces rapidly (4).

Platelet adhesion to the subendothelium is represented by the term $k_{adh}(\vec{x})\{P_{\max} - P^{se,a}\}P^{m,u(a)}$, where $k_{adh}(\vec{x})$ represents the position-dependent rate constant for platelet adherence to subendothelium. We used a position-dependent platelet adhesion rate constant to account for the initial hindrance in firm binding by collagen, as well as for the detachment of adhered platelets from the thrombogenic surface (7-9). The adhesion rate constant, $k_{adh}(\vec{x})$, is assumed to decrease as wall shear rate increases (Table S5), because the motion of the platelets transported by the blood flow could impede their firm binding to the thrombogenic surface (2). Platelet detachment from the subendothelium, as well as from bound activated platelets, is described by the term $k_{\det}(\vec{x})P^{se(b),a}$, where the detachment rate constant $k_{\det}(\vec{x})$ is proportional to the wall shear rate (Table S5). In our model, both thrombin and ADP activate platelets, as reflected by the terms $A_1(e_2)$ and $A_2([ADP])$, respectively (brackets designate concentration), in Eqs. S8 and S9. In these equations, the rate functions $A_1(e_2)$ and $A_2([ADP])$ each have the form $k^{c}_{pla}\, c/c^* + c$, where c is the agonist (i.e., thrombin or ADP) concentration and the values of k^{pla}_{c} are available in Table S5 (2,4).

The binding of mobile activated platelets with bound platelets in their close proximity is modeled by the term $k_{coh}g(\eta)P_{\max}P^{m,a}$ (Eqs. S9 and S10), where k_{coh} is the rate of platelet-platelet coherence (2,4). The proximity of an activated platelet to other bound platelets is described by the function $\eta(\vec{x},t)$ (representing a "virtual substance"), which is required to satisfy Eq. S12 (4). Here, $\vec{x}$ determines the proximity of a mobile active platelet species to the bound platelet species. The rate of binding of activated platelets to the bound platelets is proportional to their own concentration and the binding affinity function $g(\eta)$, which increases rapidly as η increases (4). In Eq. S12, the diffusion coefficient, D_η, was chosen to have the same value as the diffusion coefficient for platelets, $D^b_{platelets}$ (Table S1). The rate, ξ, at which the virtual substance η is produced, was computed so that $(D_\eta / \xi)^{1/2}$ was comparable to the typical platelet diameter of 2 µm (2).

ADP release

The ADP released from platelets moves through blood plasma and activates other platelets. The ADP kinetics in our model is governed by Eq. S13 (2,4). In Eq. S13, the source term $\sigma_{release}$ defines the release of ADP from activated platelets. This term is defined in Eq. S14, where $\hat{A}R(\tau)$ is the rate of ADP release by the newly activated and bound platelets at location x within the time interval $(\vec{x},t-\tau)d\tau$, which is described by the term $\partial/\partial t\ (P^{b,a} + P^{se,a})(\vec{x},t-\tau)d\tau$ in Eq.

S13. For the first second after the activation of a platelet, $R(\tau)$ is equal to zero, and from 1 to 5 seconds, $R(\tau)$ follows a bell-shaped curve with a peak value at 3 s (4,10). The total amount of ADP released by an activated platelet, $\hat{A}$, was set to have a value of 2.5×10^{-11} micromoles per platelet (2,4).

Biochemical reactions

The biochemical reactions leading to the generation of thrombin and fibrin take place in blood plasma, on the subendothelium, and at the binding sites located on the platelet surfaces (Eqs. S15-S62). We follow the same notation as in (4). In this notation, z_i and e_i designate the plasma concentrations of the inactive and active, respectively, forms of the protein *i*. Moreover, z_i^{se} and e_i^{se} denote the concentrations of the inactive and active, respectively, forms of the protein *i* bound to the subendothelium. Furthermore, z_i^{m} and e_i^{m} refer to the concentrations of inactive and active, respectively, forms of the protein *i* bound to platelets. Finally, the concentrations of complexes formed, e.g., by protein Z_i and protein E_j (i.e., a zymogen and an active enzyme, respectively) are denoted as $[Z_i : E_j]$. Intrinsic tenase (i.e., VIIIa:IXa) and prothrombinase (i.e., Va:Xa) complexes are denoted by *TEN* and *PRO*, respectively. TF is tissue factor that initiates the reactions leading to thrombin generation. TFPI designates tissue factor pathway inhibitor, and TFPIa is the complex TFPI:Xa. Activated protein C (*APC*) is a part of the model, but its concentration in the current study was zero, because microfluidic channel walls do not harbor thrombomodulin, which is necessary for APC formation.

The biochemical reactions on the platelet surfaces depend on the availability of free binding sites, which is described by the term $(N_i^b P^{b,a} + N_i^{se,a} P^{se,a} - z_i^{mtot} - e_i^{mtot})$ in Eqs. S21-S30. There, N_i^b and $N_i^{se,a}$ represent the total platelet surface binding site numbers, and the quantities z_i^{mtot} and e_i^{mtot} denote the concentrations of inactive and active, respectively, forms of a protein bound to the binding sites on the platelet surfaces. The variable e_2^m (platelet-bound thrombin) is the variable whose values were plotted in our figures representing thrombin generation, because our experimental protocol was designed to measure platelet-bound thrombin and fibrin (see the Methods Section in the main text). The fibrin variable in Eq. S36 is the model output that was plotted in our figures that represent fibrin accumulation.

Model parameters

The model parameter values given in the following subsections of this Supplementary Material are the same as in the original description of our computational model (2). Many of these parameters were taken from the work of Leiderman and Fogelson (4), which provided the framework for our model development. That work, in turn, relied on the parameter values from a previous study (11), which contains a detailed description of parameter value selection based on published data.

Table S1. Brownian diffusivities and other model parameters.

Parameter	Value	Refs.
$D^b_{platelets}$	2.5×10^{-7} cm^2/s	(4)
$D^b_{proteins}$	5.0×10^{-7} cm^2/s	(4)
D^b_{adp}	5.0×10^{-6} cm^2/s	(4)
D^b_{fibrin}	1.9×10^{-7} cm^2/s	(12)
k	0.0375	(3)
η	0.8	(3)
d_{rbc}	8 μm	(13)
ϕ_b	0.4	(3)
ρ	1060 kg/m^3	(14)
μ	0.0035 kg/m·s	(15)
ϕ^b_0	0.5	(4)
$\alpha_{\max}$	6.48 N·s/mm^4	(16)
a_f	55 nm	(17)

Table S2. Designations for some essential inactive and active coagulation proteins used in the model description (other protein designations are introduced in the text above).

Inactive form	Notation	Active form	Notation
II	Z_2	IIa	E_2
V	Z_5	Va	E_5
VII	Z_7	VIIa	E_7
VIII	Z_8	VIIIa	E_8
IX	Z_9	IXa	E_9
X	Z_{10}	Xa	E_{10}
Fibrinogen	Fg	Fibrin	Fn

Table S3. Initial concentrations of the coagulation proteins in the model.

Proteins	Initial concentration (M)	Refs.
Z_2	0.84×10^{-6}	(18)
Z_5	6.00×10^{-9}	(19)
Z_7	6.00×10^{-9}	(18)
E_7	0.06×10^{-9}	(20)
Z_8	0.60×10^{-9}	(18)
Z_9	54.0×10^{-9}	(18)
Z_{10}	0.10×10^{-6}	(18)
TFPI	1.50×10^{-9}	(21)
Fg	5.40×10^{-6}	(12)

Note: The initial concentrations have been adjusted for whole blood assuming a 40% hematocrit. The initial concentrations of the biochemical species not mentioned in this table were set to zero in our simulations, except the concentration of TF, which was chosen to match the experimental conditions. Specifically, the surface densities of ~2 molecules and ~0.1 molecules TF per μm^2 corresponded to the values of ~0.33 and ~0.017 $fmol/cm^2$, which were specified at every grid node of the domain boundary representing the thrombogenic surface in the model.

Table S4. Platelet surface binding site numbers. N_{zi}^{b} and N_{ei}^{b} denote the binding site numbers for Z_i and E_i on the surfaces of $P^{m,a}$, respectively. N_{zi}^{se} and N_{ei}^{se} are defined in a similar way with respect to binding to the surfaces of $P^{se,a}$.

Proteins	Binding site numbers (this many of each kind)	Refs.
N_{z2}^{b}, N_{z2}^{se}	16000	(22)
N_{e2}^{b}, N_{e2}^{se}	2000	(23)
N_{z5}^{b}, N_{z5}^{se}	3000	(24)
N_{e5}^{b}, N_{e5}^{se}	3000	(24)
N_{z8}^{b}, N_{z8}^{se}	650	(25)
N_{e8}^{b}, N_{e8}^{se}	1200	(25)
N_{z9}^{b}, N_{z9}^{se}	250	(26)
N_{e9}^{b}, N_{e9}^{se}	500	(26)
N_{z10}^{b}, N_{z10}^{se}	16000	(22)
N_{e10}^{b}, N_{e10}^{se}	2700	(27)

Note: Z_{10} binding site is shared with Z_2 (2).

Table S5. Rate constants for platelet transformations (2). **NOTE: k^0_{adh} is the parameter that was changed during model calibration; its post-calibration value is 4-fold higher than the value in the table (see main text).**

Platelet transformation	Value (units)	Refs.
$P^{m,u} \leftrightarrows P^{se,a}$	$k_{adh}(\vec{x}) = k^0_{adh} - \beta\dot{\gamma}_w \ (M^{-1} \cdot s^{-1})$	(8)
	$k_{\mathrm{det}} = \delta\dot{\gamma}_w \ (s^{-1})$	(8)
	$k^0_{adh} = 1\times10^{10} \ (M^{-1} \cdot s^{-1})$	(4)
	$\beta = \beta_0 / P_{\max} = 2\times10^5 \ (M^{-1})$<br>$\delta = 10^{-3}$	(4,8)
$P^{b,a} \rightarrow P^{se,a}$	$k_{adh}(\vec{x})$ [as above]	
$P^{m,a} \leftrightarrows P^{b,a}$	$k_{coh} \times P_{\max}$ = $1\times10^4 \ (s^{-1})$	(4)
$P^{m,a} \rightarrow P^{se,a}$	$k_{adh}(\vec{x})$ [as above]	
$P^{m,u} \rightarrow P^{m,a}$	$k^{pla}_{e_2} = 0.50\,(s^{-1})$, $k^{pla}_{adp} = 0.34\,(s^{-1})$	(4)

Table S6. Rate constants for the coagulation protein binding to platelet surfaces. The notation P_i denotes the binding sites on activated platelet surfaces for the corresponding proteins, and the notation ⇆ represents reversible binding. All the rate-constant values were taken from Refs. (4,11).

Reactions	Binding ($M^{-1} \cdot s^{-1}$)	Unbinding (s^{-1})
$Z_9 + P_9 \leftrightarrows Z_9^m$	$k_9^{on} = 1.0\times10^7$	$k_9^{off} = 2.5\times10^{-2}$
$E_9 + P_9 \leftrightarrows E_9^m$	$k_9^{on} = 1.0\times10^7$	$k_9^{off} = 2.5\times10^{-2}$
$Z_{10} + P_{10} \leftrightarrows Z_{10}^m$	$k_{10}^{on} = 1.0\times10^7$	$k_{10}^{off} = 2.5\times10^{-2}$
$E_{10} + P_{10} \leftrightarrows E_{10}^m$	$k_{10}^{on} = 1.0\times10^7$	$k_{10}^{off} = 2.5\times10^{-2}$
$Z_5 + P_5 \leftrightarrows Z_5^m$	$k_5^{on} = 5.7\times10^7$	$k_5^{off} = 0.17$
$E_5 + P_5 \leftrightarrows E_5^m$	$k_5^{on} = 5.7\times10^7$	$k_5^{off} = 0.17$
$Z_8 + P_8 \leftrightarrows Z_8^m$	$k_8^{on} = 5.0\times10^7$	$k_8^{off} = 0.17$
$E_8 + P_8 \leftrightarrows E_8^m$	$k_8^{on} = 5.0\times10^7$	$k_8^{off} = 0.17$
$Z_2 + P_2 \leftrightarrows Z_2^m$	$k_2^{on} = 1.0\times10^7$	$k_2^{off} = 5.9$
$E_2 + P_2 \leftrightarrows E_2^m$	$k_2^{on} = 1.0\times10^7$	$k_2^{off} = 5.9$

Table S7. Rate constants for the biochemical reactions (Eqs. S15-S62). The notation ⇆ represents a reversible reaction with k^+ and k^- as its forward and reverse rate constants, respectively. This reaction results in the formation of an enzyme-substrate complex, designated with ":". The complex, in turn, is converted to a product, which depends on the catalytic constant k^{cat}. The forward reaction is denoted by a single arrow (→). In Eqs. S17-S62, the rate constants are subscripted with the corresponding substrate-enzyme complex. The notation ⇆ represents reversible binding with k^+ and k^- representing the association and disassociation rate constants, respectively. **NOTE: in the biochemistry equations given below, the rate constants have subscripts designating the reactants. In this table, these subscripts are omitted.**

Sub-endothelial reactions	k^+ (M^{-1}·s^{-1})	k^- (s^{-1})	k^{cat} (s^{-1})
$E_{10} + Z_7^{se} \rightarrow E_{10} + E_7^{se}$	5.00×10^6		
$E_2 + Z_7^{se} \rightarrow E_2 + E_7^{se}$	3.92×10^5		
$E_7^{se} + Z_{10} \leftrightarrows Z_{10} : E_7^{se} \rightarrow E_7^{se} + E_{10}$	8.95×10^5	1.0	1.15
$E_7^{se} + Z_9 \leftrightarrows Z_9 : E_7^{se} \rightarrow E_7^{se} + E_9$	8.95×10^5	1.0	1.15
$TFPIa + E_7^{se} \leftrightarrows TFPIa : E_7^{se}$	1.00×10^7	1.1×10^{-3}	
**Platelet surface reactions**	k^+ (M^{-1}·s^{-1})	k^- (s^{-1})	k^{cat} (s^{-1})
$E_{10}^m + Z_5^m \rightarrow E_{10}^m + E_5^m$	1.00×10^8		
$E_2^m + Z_5^m \rightarrow E_2^m + E_5^m$	1.73×10^7		
(*) $APC + E_5^m \leftrightarrows APC : E_5^m$	6.32×10^7	1.0	
$E_{10}^m + Z_8^m \rightarrow E_{10}^m + E_8^m$	5.10×10^7		
$E_2^m + Z_8^m \rightarrow E_2^m + E_8^m$	2.64×10^7		
$TEN + Z_{10} \leftrightarrows Z_{10} : TEN \rightarrow TEN + E_{10}^m$	1.31×10^8	1.0	20.0
$PRO + Z_2 \leftrightarrows Z_2 : PRO \rightarrow PRO + E_2^m$	1.03×10^8	1.0	30.0
$E_8^m + E_9^m \leftrightarrows E_8^m : E_9^m$	1.00×10^8	0.01	

$E_5^m + E_{10}^m \leftrightarrows E_5^m : E_{10}^m$	1.00×10^{8}	0.01	
Reactions in blood plasma	k^+ ($M^{-1}\cdot s^{-1}$)	k^- (s^{-1})	k^{cat} (s^{-1})
$E_{10} + Z_7 \leftrightarrows Z_7 : E_{10} \rightarrow E_{10} + E_7$	5.00×10^{6}	1.0	5.0
$E_2 + Z_7 \leftrightarrows Z_7 : E_2 \rightarrow E_2 + E_7$	3.92×10^{5}	1.0	6.1×10^{-2}
(**) $E_{10} + Z_2 \rightarrow E_{10} + E_2$	7.5×10^{3}		
$E_2 + Z_5 \rightarrow E_2 + E_5$	1.73×10^{7}		
$E_2 + Z_8 \rightarrow E_2 + E_8$	2.64×10^{7}		
$E_{10} + TFPI \leftrightarrows E_{10} : TFPI$ (formation of *TFPIa*)	1.6×10^{7}	3.3×10^{-4}	
(***) $E_2^m + Fg \rightarrow E_2^m + Fn$	1.16×10^{7}		

All the rate-constant values were taken from Ref. (4), except the rates marked with (*), (**), and (***), which were based on Refs. (28), (29), and (2,12), respectively.

Table S8. Rate constants for inhibitory reactions by the action of antithrombin. All the rate-constant values were taken from Ref. (4).

Inhibitory reaction	Value (s^{-1})
$IXa \rightarrow IXa_{inactive}$	$k_9^{in} = 0.1$
$Xa \rightarrow Xa_{inactive}$	$k_{10}^{in} = 0.1$
$IIa \rightarrow IIa_{inactive}$	$k_2^{in} = 0.1$

These pseudo-first order reactions are used to reflect the deactivation of enzymes due to binding with antithrombin, which is assumed to be present in excess.

Model parameter sensitivities

Previously, we used our computational model to analyze the influence of several essential model parameters on clot formation (2). Specifically, we showed how our model responded to changes in the fibrinogen concentration. Moreover, we reduced the concentrations of coagulation proteins and the hematocrit level to represent dilution. Furthermore, we investigated how supplementing several coagulation proteins (namely, fibrinogen and factors II, IX, and X) affects clot formation under dilution. In those simulations, the model was stable and demonstrated robust behavior. In the present study, we considered flow conditions and k_{adh} values that are different from those in our previous studies (2,30). Taken together, our model simulation results indicate robust behavior of the model under variations in parameter values and simulation conditions.

The kinetic parameters of the biochemical reactions represented in the model have been derived from *in-vitro* studies (4,11). The rate constant values for a given biochemical reaction can vary 2-3 fold depending on the measuring laboratory and the experimental method used (31). Thus, the uncertainty in the parameter values for biochemical kinetic models can be considerable. Yet, the results of the present study, as well as our previous results for venous-shear flows (2,30), demonstrated that the default parameter set allowed the model to capture major trends in the experimental data. This may serve as a further testament to our model's robustness, which implies that parameter variations have a moderate influence on the model's outputs.

Sensitivity analysis studies using *in-vitro* models of thrombin generation suggested that the most influential biochemical parameters are the rate constants characterizing the reactions involving tissue factor and factors VII and VIIa (32,33). While the underlying biochemical model (29) is somewhat different from the one used in our fully coupled CFD model, it represents the same basic biochemistry of clot formation. Therefore, the reactions on the subendothelium – and the corresponding rate constant values – are expected to have a substantial influence on the model outputs.

Platelet transport and transformation equations

$$\frac{\partial P^{m,u}}{\partial t} = -\nabla \cdot \{W(\varphi_t)(\vec{u}P^{m,u} - D\nabla \psi^{m,u})\} \\ -k_{adh}(\vec{x})\{P_{\max} - P^{se,a}\}P^{m,u} - \{A_1(e_2) + A_2([ADP])\}P^{m,u}, \tag{S8}$$

$$\frac{\partial P^{m,a}}{\partial t} = -\nabla \cdot \{W(\varphi_t)(\vec{u}P^{m,a} - D\nabla \psi^{m,a})\} - k_{adh}(\vec{x})\{P_{\max} - P^{se,a}\}P^{m,a} \\ +\{A_1(e_2) + A_2([ADP])\}P^{m,u} - k_{coh}g(\eta)P_{\max}P^{m,a} + k_{\det}(\vec{x})P^{b,a} + k_{\det}(\vec{x})P^{se,a}, \tag{S9}$$

$$\frac{\partial P^{b,a}}{\partial t} = -k_{adh}(\vec{x})\{P_{\max} - P^{se,a}\}P^{b,a} + k_{coh}g(\eta)P_{\max}P^{m,a} - k_{\det}(\vec{x})P^{b,a}, \tag{S10}$$

$$\frac{\partial P^{se,a}}{\partial t} = k_{adh}(\vec{x})\{P_{\max} - P^{se,a}\}(P^{m,a} + P^{m,u} + P^{b,a}) - k_{\det}(\vec{x})P^{se,a} \ , \tag{S11}$$

$$\frac{\partial \eta}{\partial t} = D_\eta \Delta \eta - \xi \eta + \xi \left(\frac{P^{b,a} + P^{se,a}}{P_{\max}} \right). \tag{S12}$$

ADP release by activated platelets

$$\frac{\partial [ADP]}{\partial t} = -\vec{u} \cdot \nabla [ADP] + \nabla \cdot (D\nabla [ADP]) + \sigma_{release} \, , \tag{S13}$$

$$\sigma_{release}(\vec{x},t) = \int_0^\infty \hat{A}R(\tau) \frac{\partial}{\partial t}(P^{b,a} + P^{se,a})(\vec{x}, t-\tau)d\tau. \tag{S14}$$

Biochemical reactions that occur on the sub-endothelium

$$\frac{\partial z_7^{se}}{\partial t} = k_7^{on} z_7([TF] - e_7^{setot} - z_7^{setot}) - k_7^{off} z_7^{se} - k^+_{z_7^{se}:e_{10}} z_7^{se} e_{10} - k^+_{z_7^{se}:e_2} z_7^{se} e_2 \\ - k_{adh}(\vec{x}) z_7^{se} \left(P^{m,a} + P^{m,u} + P^{b,a}\right), \tag{S15}$$

$$\frac{\partial e_7^{se}}{\partial t} = k_7^{on} e_7([TF] - e_7^{setot} - z_7^{setot}) - k_7^{off} e_7^{se} + k^+_{z_7^{se}:e_{10}} z_7^{se} e_{10} + k^+_{z_7^{se}:e_2} z_7^{se} e_2 \\ + \left(k^-_{z_{10}:e_7^{se}} + k^{cat}_{z_{10}:e_7^{se}}\right) \left[Z_{10} : E_7^{se}\right] - k^+_{z_{10}:e_7^{se}} z_{10} e_7^{se} + \left(k^-_{z_9:e_7^{se}} + k^{cat}_{z_9:e_7^{se}}\right) \left[Z_9 : E_7^{se}\right] - k^+_{z_9:e_7^{se}} z_9 e_7^{se} \\ -k^+_{tfpia:e_7^{se}} \left[TFPIa\right] e_7^{se} + k^-_{tfpia:e_7^{se}} \left[TFPIa : E_7^{se}\right] - k_{adh}(\vec{x}) e_7^{se} \left(P^{m,a} + P^{m,u} + P^{b,a}\right) \ , \tag{S16}$$

$$\frac{\partial\left[Z_9 : E_7^{se}\right]}{\partial t} = k^{+}_{z_9:e_7^{se}} z_9 e_7^{se} - \left(k^{-}_{z_9:e_7^{se}} + k^{cat}_{z_9:e_7^{se}}\right)\left[Z_9 : E_7^{se}\right] - k_{adh}(\vec{x})[Z_9 : E_7^{se}]\left(P^{m,a} + P^{m,u} + P^{b,a}\right), \qquad \text{(S17)}$$

$$\frac{\partial\left[Z_{10} : E_7^{se}\right]}{\partial t} = k^{+}_{z10:e_7^{se}} z_{10} e_7^{se} - \left(k^{-}_{z_{10}:e_7^{se}} + k^{cat}_{z10:e_7^{se}}\right)\left[Z_{10} : E_7^{se}\right]$$
$$- k_{adh}(\vec{x})[Z_{10} : E_7^{se}]\left(P^{m,a} + P^{m,u} + P^{b,a}\right), \qquad \text{(S18)}$$

$$\frac{\partial\left[TFPIa : E_7^{se}\right]}{\partial t} = k^{+}_{TFPIa:e_7^{se}}[TFPIa]e_7^{se} - k^{-}_{TFPIa:e_7^{se}}\left[TFPIa : E_7^{se}\right]$$
$$-k_{adh}(\vec{x})[TFPIa : E_7^{se}]\left(P^{m,a} + P^{m,u} + P^{b,a}\right) , \qquad \text{(S19)}$$

$$\frac{\partial[TF]}{\partial t} = -k_{adh}(\vec{x})[TF]\left(P^{m,a} + P^{m,u} + P^{b,a}\right). \qquad \text{(S20)}$$

Biochemical reactions that occur on platelet surfaces

$$\frac{\partial z_2^m}{\partial t} = k_2^{on} z_2 (N_{z2}^b P^{b,a} + N_{z2}^{se} P^{se,a} - z_2^{mtot} - e_2^{mtot}) - k_2^{off} z_2^m$$
$$- k^{+}_{z_2^m:PRO} z_2^m [PRO] + k^{-}_{z_2^m:PRO}[Z_2^m : PRO], \qquad \text{(S21)}$$

$$\frac{\partial e_2^m}{\partial t} = k_2^{on} e_2 (N_{e2}^b P^{b,a} + N_{e2}^{se} P^{se,a} - z_2^{mtot} - e_2^{mtot}) - k_2^{off} e_2^m + k^{cat}_{z_2^m:PRO}[Z_2^m : PRO] - k^{+}_{z_5^m:e_2^m} z_5^m e_2^m$$
$$-k^{+}_{z_8^m:e_2^m} z_8^m e_2^m , \qquad \text{(S22)}$$

$$\frac{\partial z_5^m}{\partial t} = k_5^{on} z_5 (N_{z5}^b P^{b,a} + N_{z5}^{se} P^{se,a} - z_5^{mtot} - e_5^{mtot}) - k_5^{off} z_5^m - k^{+}_{z_5^m:e_{10}^m} z_5^m e_{10}^m - k^{+}_{z_5^m:e_2^m} z_5^m e_2^m , \qquad \text{(S23)}$$

$$\frac{\partial e_5^m}{\partial t} = k_5^{on} e_5 (N_{e5}^b P^{b,a} + N_{e5}^{se} P^{se,a} - z_5^{mtot} - e_5^{mtot}) - k_5^{off} e_5^m + k^{+}_{z_5^m:e_{10}^m} z_5^m e_{10}^m + k^{+}_{z_5^m:e_2^m} z_5^m e_2^m$$
$$+ k^{-}_{pro}[PRO] - k^{+}_{pro} e_5^m e_{10}^m - k^{+}_{apc:e_5^m}[APC]e_5^m + k^{-}_{apc:e_5^m}[APC : E_5^m], \qquad \text{(S24)}$$

$$\frac{\partial z_8^m}{\partial t} = k_8^{on} z_8 (N_{z8}^b P^{b,a} + N_{z8}^{se} P^{se,a} - z_8^{mtot} - e_8^{mtot}) - k_8^{off} z_8^m - k^{+}_{z_8^m:e_{10}^m} z_8^m e_{10}^m - k^{+}_{z_8^m:e_2^m} z_8^m e_2^m , \qquad \text{(S25)}$$

$$\frac{\partial e_8^m}{\partial t} = k_8^{on} e_8 (N_{e8}^b P^{b,a} + N_{e8}^{se} P^{se,a} - z_8^{mtot} - e_8^{mtot}) - k_8^{off} e_8^m + k^{+}_{z_8^m:e_{10}^m} z_8^m e_{10}^m + k^{+}_{z_8^m:e_2^m} z_8^m e_2^m$$
$$+k^{-}_{ten}[TEN] - k^{+}_{ten} e_8^m e_9^m , \qquad \text{(S26)}$$

$$\frac{\partial z_9^m}{\partial t} = k_9^{on} z_9 (N_{z9}^b P^{b,a} + N_{z9}^{se} P^{se,a} - z_9^{mtot} - e_9^{mtot}) - k_9^{off} z_9^m, \tag{S27}$$

$$\frac{\partial e_9^m}{\partial t} = k_9^{on} e_9 (N_{e9}^b P^{b,a} + N_{e9}^{se} P^{se,a} - z_9^{mtot} - e_9^{mtot}) - k_9^{off} e_9^m + k_{ten}^- [TEN] - k_{ten}^+ e_8^m e_9^m, \tag{S28}$$

$$\frac{\partial z_{10}^m}{\partial t} = k_{10}^{on} z_{10} (N_{z10}^b P^{b,a} + N_{z10}^{se} P^{se,a} - z_{10}^{mtot} - e_{10}^{mtot}) - k_{10}^{off} z_{10}^m + k_{z_{10}^m:ten}^- [Z_{10}^m : TEN] - k_{z_{10}^m:ten}^+ z_{10}^m [TEN], \tag{S29}$$

$$\frac{\partial e_{10}^m}{\partial t} = k_{10}^{on} e_{10} (N_{e10}^b P^{b,a} + N_{e10}^{se} P^{se,a} - z_{10}^{mtot} - e_{10}^{mtot}) - k_{10}^{off} e_{10}^m - k_{z_5^m:e_{10}^m}^+ z_5^m e_{10}^m - k_{z_8^m:e_{10}^m}^+ z_8^m e_{10}^m + k_{pro}^- [PRO] - k_{pro}^+ e_5^m e_{10}^m + k_{z_{10}^m:ten}^{cat} [Z_{10}^m : TEN], \tag{S30}$$

$$\frac{\partial [TEN]}{\partial t} = k_{ten}^+ e_8^m e_9^m - k_{ten}^- [TEN] - k_{z_{10}^m:ten}^+ z_{10}^m [TEN] + (k_{z_{10}^m:ten}^{cat} + k_{z_{10}^m:ten}^-)[Z_{10}^m : TEN], \tag{S31}$$

$$\frac{\partial [PRO]}{\partial t} = k_{pro}^+ e_5^m e_{10}^m - k_{pro}^- [PRO] - k_{z_2^m:pro}^+ z_2^m [PRO] + (k_{z_2^m:pro}^{cat} + k_{z_2^m:pro}^-)[Z_2^m : PRO], \tag{S32}$$

$$\frac{\partial [Z_2^m : PRO]}{\partial t} = k_{z_2^m:pro}^+ z_2^m [PRO] - (k_{z_2^m:pro}^{cat} + k_{z_2^m:pro}^-)[Z_2^m : PRO], \tag{S33}$$

$$\frac{\partial [Z_{10}^m : TEN]}{\partial t} = k_{z_{10}^m:ten}^+ z_{10}^m [TEN] - (k_{z_{10}^m:ten}^{cat} + k_{z_{10}^m:ten}^-)[Z_{10}^m : TEN], \tag{S34}$$

$$\frac{\partial [APC : E_5^m]}{\partial t} = k_{apc:e_5^m}^+ e_5^m [APC] - (k_{apc:e_5^m}^{cat} + k_{apc:e_5^m}^-)[APC : E_5^m], \tag{S35}$$

$$\frac{\partial [Fn]}{\partial t} = \nabla \cdot (D \nabla [Fn]) + k_{Fg:e_2^m}^+ [Fg][e_2^m]. \tag{S36}$$

Biochemical reactions that take place in blood plasma

$$\frac{\partial z_2}{\partial t} = -\nabla \cdot (\vec{u} z_2 - D\nabla z_2) - k_2^{on} z_2 (N_{z2}^{b} P^{b,a} + N_{z2}^{se} P^{se,a} - z_2^{mtot} - e_2^{mtot}) - k_{z_2:e_{10}}^{+} z_2 e_{10} + k_2^{off} z_2^{m}, \quad \text{(S37)}$$

$$\frac{\partial e_2}{\partial t} = -\nabla \cdot (\vec{u} e_2 - D\nabla e_2) - k_2^{on} e_2 (N_{e2}^{b} P^{b,a} + N_{e2}^{se} P^{se,a} - z_2^{mtot} - e_2^{mtot}) + k_2^{off} e_2^{m} - k_{z5:e2}^{+} z_5 e_2 - k_{z8:e2}^{+} z_8 e_2 + k_{z2:e10}^{+} z_2 e_{10} + (k_{z7:e2}^{-} + k_{z7:e2}^{cat})[Z_7 : E_2] - k_{z7:e2}^{+} z_7 e_2 - k_2^{in} e_2, \quad \text{(S38)}$$

$$\frac{\partial z_5}{\partial t} = -\nabla \cdot (\vec{u} z_5 - D\nabla z_5) - k_5^{on} z_5 (N_{z5}^{b} P^{b,a} + N_{z5}^{se} P^{se,a} - z_5^{mtot} - e_5^{mtot}) + k_5^{off} z_5^{m} - k_{z_5:e_2}^{+} z_5 e_2 + N_5 \frac{\partial (P^{b,a} + P^{se,a})}{\partial t}, \quad \text{(S39)}$$

$$\frac{\partial e_5}{\partial t} = -\nabla \cdot (\vec{u} e_5 - D\nabla e_5) - k_9^{on} e_5 (N_{e5}^{b} P^{b,a} + N_{e5}^{se} P^{se,a} - z_5^{mtot} - e_5^{mtot}) + k_5^{off} e_5^{m} + k_{z_5:e_2}^{+} z_5 e_2, \quad \text{(S40)}$$

$$\frac{\partial z_7}{\partial t} = -\nabla \cdot (\vec{u} z_7 - D\nabla z_7) - k_{z_7:e_{10}}^{+} z_7 e_{10} + k_{z_7:e_{10}}^{-} [Z_7 : E_{10}] + k_{z_7:e_2}^{-} [Z_7 : E_2] - k_{z_7:e_2}^{+} z_7 e_2, \quad \text{(S41)}$$

$$\frac{\partial e_7}{\partial t} = -\nabla \cdot (\vec{u} e_7 - D\nabla e_7) + k_{z_7:e_{10}}^{cat} [Z_7 : E_{10}] + k_{z_7:e_2}^{cat} [Z_7 : E_2], \quad \text{(S42)}$$

$$\frac{\partial z_8}{\partial t} = -\nabla \cdot (\vec{u} z_8 - D\nabla z_8) - k_8^{on} z_8 (N_{z8}^{b} P^{b,a} + N_{z8}^{se} P^{se,a} - z_8^{mtot} - e_8^{mtot}) + k_8^{off} z_8^{m} - k_{z_8:e_2}^{+} z_8 e_2, \quad \text{(S43)}$$

$$\frac{\partial e_8}{\partial t} = -\nabla \cdot (\vec{u} e_8 - D\nabla e_8) - k_8^{on} e_8 (N_{e8}^{b} P^{b,a} + N_{e8}^{se} P^{se,a} - z_8^{mtot} - e_8^{mtot}) + k_8^{off} e_8^{m} + k_{z_8:e_2}^{+} z_8 e_2, \quad \text{(S44)}$$

$$\frac{\partial z_9}{\partial t} = -\nabla \cdot (\vec{u} z_9 - D\nabla z_9) - k_9^{on} z_9 (N_{z9}^{b} P^{b,a} + N_{z9}^{se} P^{se,a} - z_9^{mtot} - e_9^{mtot}) + k_9^{off} z_9^{m}, \quad \text{(S45)}$$

$$\frac{\partial e_9}{\partial t} = -\nabla \cdot (\vec{u} e_9 - D\nabla e_9) - k_9^{on} e_9 (N_{e9}^{b} P^{b,a} + N_{e9}^{se} P^{se,a} - z_9^{mtot} - e_9^{mtot}) + k_9^{off} e_9^{m} - k_9^{in} e_9, \quad \text{(S46)}$$

$$\frac{\partial z_{10}}{\partial t} = -\nabla \cdot (\vec{u} z_{10} - D\nabla z_{10}) - k_{10}^{on} z_{10} (N_{z10}^{b} P^{b,a} + N_{z10}^{se} P^{se,a} - z_{10}^{mtot} - e_{10}^{mtot}) + k_{10}^{off} z_{10}^{m} \ , \tag{S47}$$

$$\begin{aligned}\frac{\partial e_{10}}{\partial t} = &-\nabla \cdot (\vec{u} e_{10} - D\nabla e_{10}) - k_{10}^{on} e_{10} (N_{e10}^{b} P^{b,a} + N_{e10}^{se} P^{se,a} - z_{10}^{mtot} - e_{10}^{mtot}) + k_{10}^{off} e_{10}^{m} \\ &- k_{tfpia:e_{10}}^{+} [TFPI] e_{10} + k_{tfpia:e_{10}}^{-} [TFPIa] + (k_{z_7:e_{10}}^{-} + k_{z_7:e_{10}}^{cat}) [Z_7 : E_{10}] - k_{z_7:e_{10}}^{+} z_7 e_{10} - k_{10}^{in} e_{10} \ ,\end{aligned} \tag{S48}$$

$$\frac{\partial [Z_7 : E_{10}]}{\partial t} = -\nabla \cdot (\vec{u} [Z_7 : E_{10}] - D\nabla [Z_7 : E_{10}]) + k_{z_7:e_{10}}^{+} z_7 e_{10} - (k_{z_7:e_{10}}^{cat} + k_{z_7:e_{10}}^{-}) [Z_7 : E_{10}] , \tag{S49}$$

$$\frac{\partial [Z_7 : E_2]}{\partial t} = -\nabla \cdot (\vec{u} [Z_7 : E_2] - D\nabla [Z_7 : E_2]) + k_{z_7:e_2}^{+} z_7 e_2 - (k_{z_7:e_2}^{cat} + k_{z_7:e_2}^{-}) [Z_7 : E_2] , \tag{S50}$$

$$\frac{\partial [TFPI]}{\partial t} = -\nabla \cdot (\vec{u} [TFPI] - D\nabla [TFPI]) - k_{tfpia:e_{10}}^{+} [TFPI] e_{10} + k_{tfpia:e_{10}}^{-} [TFPIa] \ , \tag{S51}$$

$$\frac{\partial [TFPIa]}{\partial t} = -\nabla \cdot (\vec{u} [TFPIa] - D\nabla [TFPIa]) + k_{tfpia:e_{10}}^{+} [TFPI] e_{10} - k_{tfpia:e_{10}}^{-} [TFPIa] , \tag{S52}$$

$$\begin{aligned}\frac{\partial [APC]}{\partial t} = &-\nabla \cdot (\vec{u} [APC] - D\nabla [APC]) + (k_{apc:e_5^m}^{cat} + k_{apc:e_5^m}^{-}) [APC : E_5^m] \\ &- k_{apc:e_5^m}^{+} e_5^m [APC] ,\end{aligned} \tag{S53}$$

$$\frac{\partial [Fg]}{\partial t} = -\nabla \cdot (\vec{u} [Fg] - D\nabla [Fg]) - k_{Fg:e_2^m}^{+} [Fg] [e_2^m] . \tag{S54}$$

Boundary conditions for the convection-diffusion-reaction equation (these conditions characterize the reactions on the sub-endothelium)

$$-D \frac{\partial e_2}{\partial y} = -k_{z_7^{se}:e_2}^{+} z_7^{se} e_2 \ , \tag{S55}$$

$$-D \frac{\partial z_7}{\partial y} = -k_7^{on} z_7 ([TF] - z_7^{setot} - e_7^{setot}) + k_7^{off} z_7^{se} , \tag{S56}$$

$$-D \frac{\partial e_7}{\partial y} = -k_7^{on} e_7 ([TF] - z_7^{setot} - e_7^{setot}) + k_7^{off} e_7^{se} \ , \tag{S57}$$

$$-D\frac{\partial z_9}{\partial y} = -k^{+}_{z9:e_7^{se}} z_9 e_7^{se} + k^{-}_{z9:e_7^{se}} [Z_9 : E_7^{se}], \tag{S58}$$

$$-D\frac{\partial e_9}{\partial y} = k^{cat}_{z9:e_7^{se}} [Z_9 : E_7^{se}], \tag{S59}$$

$$-D\frac{\partial z_{10}}{\partial y} = -k^{+}_{z_{10}:e_7^{se}} z_{10} e_7^{se} + k^{-}_{z_{10}:e_7^{se}} [Z_{10} : E_7^{se}], \tag{S60}$$

$$-D\frac{\partial e_{10}}{\partial y} = -k^{+}_{z_7^{se}:e_{10}} e_{10} z_7^{se} + k^{cat}_{z_{10}:e_7^{se}} [Z_{10} : E_7^{se}], \tag{S61}$$

$$-D\frac{\partial [TFPIa]}{\partial y} = -k^{+}_{TFPIa:e_7^{se}} [TFPIa] e_7^{se} + k^{-}_{TFPIa:e_7^{se}} [TFPIa : E_7^{se}]\,. \tag{S62}$$

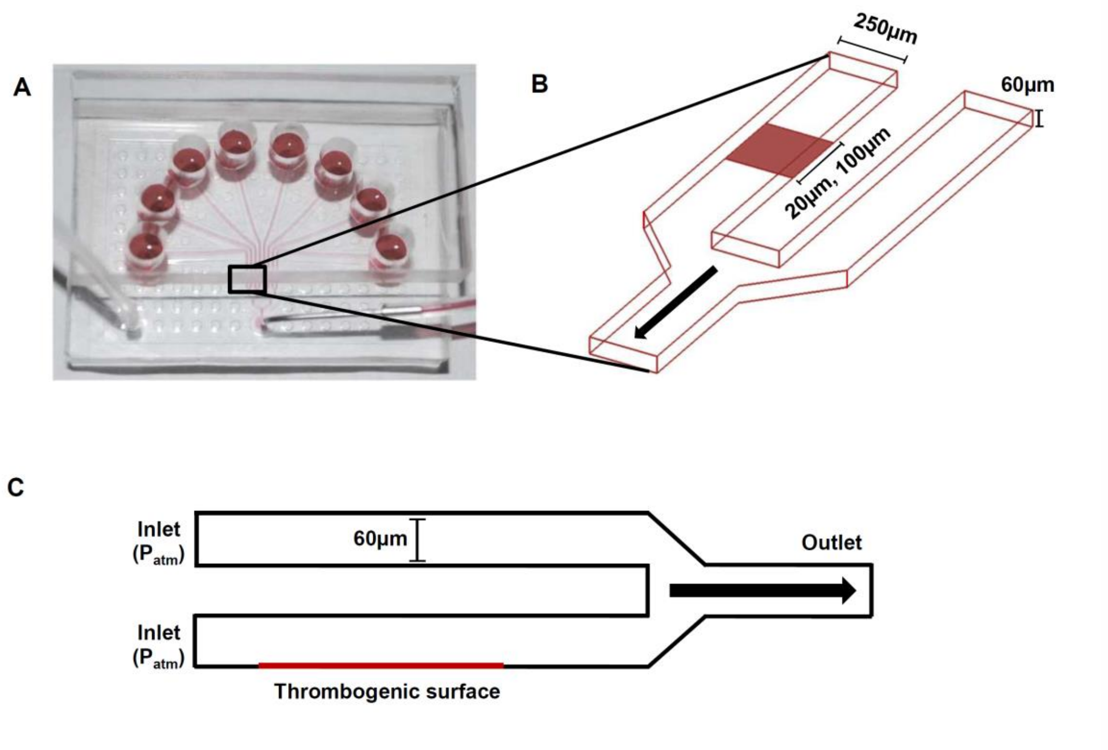

Fig. S1. The microfluidic device geometry in our experimental and computational analyses. (A) The 8-channel microfluidic device (also shown in Fig. 1). The channels have separate inlets and converge at a common outlet. The eight separate wells are the sources of the blood entering the separate channel inlets. (B) A zoom-in of a channel pair (one of the four pairs). One of the channels harbors a thrombogenic surface, and the channels are connected at the outlet. The channel dimensions are shown in the subplot. (C) The 2-D geometry representing the pair of channels shown in subplot B. Although in the actual microfluidic device the channels are positioned side by side, in the 2-D representation they are stacked on top of one another (2,30,34). The inside of the 2-D geometry defined the computational domain for our CFD simulations. This computational domain had no-slip and no-penetration boundary conditions on at the walls, a velocity boundary condition (20 µL/min) at the outlet, and (atmospheric) pressure boundary conditions at the two inlets.

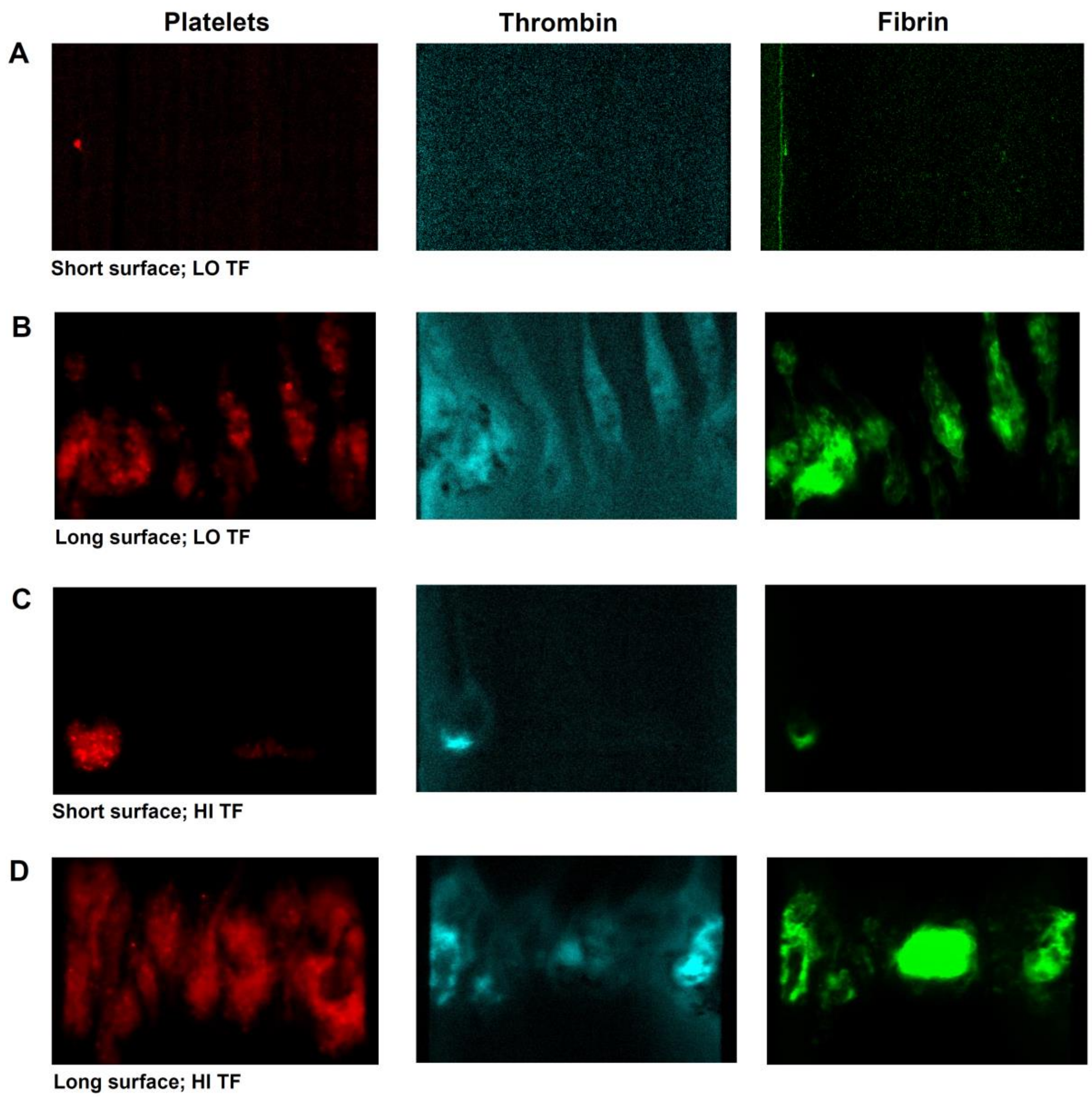

Fig. S2. Fluorescent images representing the accumulation of platelets (left), thrombin (middle), and fibrin (right) at the thrombogenic surface under the considered experimental conditions. These images correspond to one of the five donors and one repetition (i.e., representative individual clotting events) and were taken at 400 s. For each image, the horizontal dimension is the width of the microfluidic channel (i.e., 250 µm), whereas the vertical dimension corresponds to the channel length. The blood flow direction is from the bottom to the top. (A)-(D) The four experimental conditions analyzed [long or short thrombogenic surface, and high ("HI TF") or low ("LO TF") TF surface density].

REFERENCES

1. Zydney, A. L., and Colton, C. K. Augmented solute transport in the shear flow of a concentrated suspension. *PhysicoChem Hydrodyn* 1988; **10**: 77-96.
2. Govindarajan, V., Rakesh, V., Reifman, J., and Mitrophanov, A. Y. Computational study of thrombus formation and clotting factor effects under venous flow conditions. *Biophys J* 2016; **110**: 1869-85.
3. Hund, S. J., and Antaki, J. F. An extended convection diffusion model for red blood cell-enhanced transport of thrombocytes and leukocytes. *Phys Med Biol* 2009; **54**: 6415-35.
4. Leiderman, K., and Fogelson, A. L. Grow with the flow: a spatial-temporal model of platelet deposition and blood coagulation under flow. *Math Med Biol* 2011; **28**: 47-84.
5. Davies, C. N. The separation of airborne dust and particles. *Arhiv za Higijenu Rada* 1950; **1**: 393-427.
6. Bark, D. L., Jr., and Ku, D. N. Platelet transport rates and binding kinetics at high shear over a thrombus. *Biophys J* 2013; **105**: 502-11.
7. Goodman, P. D., Barlow, E. T., Crapo, P. M., Mohammad, S. F., and Solen, K. A. Computational model of device-induced thrombosis and thromboembolism. *Ann Biomed Eng* 2005; **33**: 780-97.
8. Tokarev, A. A., Butylin, A. A., and Ataullakhanov, F. I. Platelet adhesion from shear blood flow is controlled by near-wall rebounding collisions with erythrocytes. *Biophys J* 2011; **100**: 799-808.
9. Wu, Y. P., de Groot, P. G., and Sixma, J. J. Shear-stress-induced detachment of blood platelets from various surfaces. *Arterioscler Thromb Vasc Biol* 1997; **17**: 3202-7.
10. Reed, G. L., Fitzgerald, M. L., and Polgar, J. Molecular mechanisms of platelet exocytosis: insights into the "secrete" life of thrombocytes. *Blood* 2000; **96**: 3334-42.
11. Kuharsky, A. L., and Fogelson, A. L. Surface-mediated control of blood coagulation: the role of binding site densities and platelet deposition. *Biophys J* 2001; **80**: 1050-74.
12. Neeves, K. B., Illing, D. A., and Diamond, S. L. Thrombin flux and wall shear rate regulate fibrin fiber deposition state during polymerization under flow. *Biophys J* 2010; **98**: 1344-52.
13. Zhang, J., Johnson, P. C., and Popel, A. S. Red blood cell aggregation and dissociation in shear flows simulated by lattice Boltzmann method. *J Biomech* 2008; **41**: 47-55.
14. Jafari, A., Mousavi, S. M., and Kolari, P. Numerical investigation of blood flow. Part I: In microvessel bifurcations. *Commun Nonlinear Sci Numer Simul* 2008; **13**: 1615-26.
15. Papaioannou, T. G., and Stefanadis, C. Vascular wall shear stress: basic principles and methods. *Hellenic J Cardiol* 2005; **46**: 9-15.
16. Adolph, R., Vorp, D. A., Steed, D. L., Webster, M. W., Kameneva, M. V., and Watkins, S. C. Cellular content and permeability of intraluminal thrombus in abdominal aortic aneurysm. *J Vasc Surg* 1997; **25**: 916-26.
17. Wufsus, A. R., Macera, N. E., and Neeves, K. B. The hydraulic permeability of blood clots as a function of fibrin and platelet density. *Biophys J* 2013; **104**: 1812-23.
18. Mann, K. G., Nesheim, M. E., Church, W. R., Haley, P., and Krishnaswamy, S. Surface-dependent reactions of the vitamin K-dependent enzyme complexes. *Blood* 1990; **76**: 1-16.

19. Mann, K. G., Bovill, E. G., and Krishnaswamy, S. Surface-dependent reactions in the propagation phase of blood coagulation. *Ann N Y Acad Sci* 1991; **614**: 63-75.
20. Morrissey, J. H. Tissue factor modulation of factor VIIa activity: use in measuring trace levels of factor VIIa in plasma. *Thromb Haemost* 1995; **74**: 185-8.
21. Novotny, W. F., Brown, S. G., Miletich, J. P., Rader, D. J., and Broze, G. J., Jr. Plasma antigen levels of the lipoprotein-associated coagulation inhibitor in patient samples. *Blood* 1991; **78**: 387-93.
22. Scandura, J. M., Ahmad, S. S., and Walsh, P. N. A binding site expressed on the surface of activated human platelets is shared by factor X and prothrombin. *Biochemistry* 1996; **35**: 8890-902.
23. Brass, L. F., Ahuja, M., Belmonte, E., Pizarro, S., Tarver, A., and Hoxie, J. A. The human platelet thrombin receptor. Turning it on and turning it off. *Ann N Y Acad Sci* 1994; **714**: 1-12.
24. Walsh, P. N., and Schmaier, A. H. Platelet-coagulant protein interactions. *Hemostasis and Thrombosis: Basic Principles and Clinical Practice (ed 3). Philadelphia, PA, Lippincott* 1994: 629-51.
25. Ahmad, S. S., Scandura, J. M., and Walsh, P. N. Structural and functional characterization of platelet receptor-mediated factor VIII binding. *J Biol Chem* 2000; **275**: 13071-81.
26. Ahmad, S. S., Rawala-Sheikh, R., and Walsh, P. N. Comparative interactions of factor IX and factor IXa with human platelets. *J Biol Chem* 1989; **264**: 3244-51.
27. Mann, K. G., Krishnaswamy, S., and Lawson, J. H. Surface-dependent hemostasis. *Sem Hematol* 1992; **29**: 213-26.
28. Jordan, S. W., and Chaikof, E. L. Simulated surface-induced thrombin generation in a flow field. *Biophys J* 2011; **101**: 276-86.
29. Hockin, M. F., Jones, K. C., Everse, S. J., and Mann, K. G. A model for the stoichiometric regulation of blood coagulation. *J Biol Chem* 2002; **277**: 18322-33.
30. Govindarajan, V., Zhu, S., Li, R., Lu, Y., Diamond, S. L., Reifman, J., et al. Impact of tissue factor localization on blood clot structure and resistance under venous shear. *Biophys J* 2018; **114**: 978-91.
31. Bravo, M. C., Orfeo, T., Mann, K. G., and Everse, S. J. Modeling of human factor Va inactivation by activated protein C. *BMC Syst Biol* 2012; **6**: 45.
32. Danforth, C. M., Orfeo, T., Mann, K. G., Brummel-Ziedins, K. E., and Everse, S. J. The impact of uncertainty in a blood coagulation model. *Math Med Biol* 2009; **26**: 323-36.
33. Mitrophanov, A. Y., Rosendaal, F. R., and Reifman, J. Computational analysis of the effects of reduced temperature on thrombin generation: the contributions of hypothermia to coagulopathy. *Anesth Analg* 2013; **117**: 565-74.
34. Colace, T. V., Muthard, R. W., and Diamond, S. L. Thrombus growth and embolism on tissue factor-bearing collagen surfaces under flow: role of thrombin with and without fibrin. *Arterioscler Thromb Vasc Biol* 2012; **32**: 1466-76.
